# *Clumppling*: cluster matching and permutation program with integer linear programming

**DOI:** 10.1093/bioinformatics/btad751

**Published:** 2023-12-14

**Authors:** Xiran Liu, Naama M Kopelman, Noah A Rosenberg

**Affiliations:** Institute for Computational and Mathematical Engineering, Stanford University, Stanford, CA 94305, United States; Faculty of Sciences, Holon Institute of Technology, Holon 58109, Israel; Institute for Computational and Mathematical Engineering, Stanford University, Stanford, CA 94305, United States; Department of Biology, Stanford University, Stanford, CA 94305, United States

## Abstract

**Motivation:**

In the mixed-membership unsupervised clustering analyses commonly used in population genetics, multiple replicate data analyses can differ in their clustering solutions. Combinatorial algorithms assist in aligning clustering outputs from multiple replicates so that clustering solutions can be interpreted and combined across replicates. Although several algorithms have been introduced, challenges exist in achieving optimal alignments and performing alignments in reasonable computation time.

**Results:**

We present *Clumppling*, a method for aligning replicate solutions in mixed-membership unsupervised clustering. The method uses integer linear programming for finding optimal alignments, embedding the cluster alignment problem in standard combinatorial optimization frameworks. In example analyses, we find that it achieves solutions with preferred values of a desired objective function relative to those achieved by *Pong* and that it proceeds with less computation time than *Clumpak*. It is also the first method to permit alignments across replicates with multiple arbitrary values of the number of clusters *K*.

**Availability and implementation:**

*Clumppling* is available at https://github.com/PopGenClustering/Clumppling.

## 1 Introduction

Population-genetic mixed-membership unsupervised clustering methods, such as *Structure* (Pritchard *et al.* 2000) and *Admixture* (Alexander *et al.* 2009), are essential tools for understanding patterns of genetic variation in populations. These methods make use of individual genomes to infer population-genetic clusters; each individual is assigned a membership vector, in which each entry represents the inferred membership of the individual in a specific cluster.

Mixed-membership unsupervised clustering typically employs stochastic steps so that output memberships from independent runs of a clustering approach on the same set of individuals can differ. Membership differences across replicate analyses with the same settings can result from one of two sources. One source is *label-switching*, in which the clusters and the patterns of co-clustering of individuals across clusters are identical between runs, but the clusters are ordered differently across runs. The second source is *genuine multimodality*, in which the clustering algorithm yields multiple local optima that represent different patterns of co-clustering of individuals. The clustering methods are often employed with different settings, most notably for the number of clusters, denoted *K*, so that membership estimates in different runs can differ for additional reasons. Because multiple potential sources contribute to clustering differences among replicates, in mixed-membership unsupervised clustering analysis, it is important to align the clusters across these replicates—to resolve label-switching, to assess genuine multimodality with fixed settings, and to examine clustering solutions with different values of *K*.

Three main methods exist for resolving the cluster alignment across runs of population-genetic unsupervised clustering: *Clumpp* (Jakobsson and Rosenberg 2007), *Clumpak* (Kopelman *et al.* 2015), and *Pong* (Behr *et al.* 2016). The methods differ slightly in the precise cluster alignment problems they solve, the algorithmic rationale for their alignment solutions, and their computational performance. Our goal is to introduce a new method that expands the set of scenarios in which cluster alignment can be performed, connects cluster alignment in population genetics to classic techniques of combinatorial optimization, and introduces computational improvements. The new method is *Clumppling*: CLUster Matching and Permutation Program with integer Linear programmING.

## 2 Review of existing approaches

### 2.1 *Clumpp*

*Clumpp* (Jakobsson and Rosenberg 2007) aligns replicates with equally many clusters. It either enumerates all possible permutations of clusters for all replicates and returns the one that maximizes an average pairwise similarity between all replicates, or adopts a greedy algorithm to sequentially align successive replicates. The greedy algorithm reduces computational time but does not necessarily find the optimal alignment. To further speed up the alignment process, *Clumpp* has a “LargeKGreedy” method to sequentially align a replicate column-wise rather than all at once.

### 2.2 *Clumpak*

*Clumpak* (Kopelman *et al.* 2015) extends beyond *Clumpp* in two ways: algorithmic mode detection and algorithmic alignment of replicates with different numbers of clusters. First, it uses *Clumpp* to align replicates with the same *K*. It then detects modes by constructing a similarity network from pairs of aligned replicates and using the Markov clustering algorithm to define nearly identical groups of replicates in the network: modes. A mode is represented by the mean memberships of replicates in the mode.

To assess replicates with different numbers of clusters, *Clumpak* examines the alignment of modes with K+1 clusters to those with *K* clusters. It aligns replicates with K+1 and *K* clusters by including an empty cluster and solving a one-to-one matching problem.

### 2.3 *Pong*

*Pong* (Behr *et al.* 2016) views the problem of aligning pairs of replicates with equally many clusters as an *assignment problem* (Burkard *et al.* 2009). It uses the polynomial-time Hungarian algorithm for this optimization.

For mode detection among replicates with a fixed number of clusters, *Pong* follows a simple approach. It constructs a similarity network of aligned replicates and uses a user-specified threshold to remove edges, taking the disjoint cliques in the network as modes. A representative replicate is then randomly chosen from each clique to represent the mode.

To align a pair of representative replicates of the major modes with numbers of clusters *K* and K+1, *Pong* considers mergings of every possible pair of clusters from the replicate with K+1 clusters and finds the optimal alignment from all one-to-one alignments. It proceeds through consecutive *K* values to align major modes across different *K* values.

### 2.4 Improvements provided by *Clumppling*

The existing methods have been used in thousands of studies. Nevertheless, opportunities exist for more fully integrating cluster alignment methods with frameworks of combinatorial optimization and network theory, for addressing complex alignment scenarios, and for improving computation time. We highlight several desirable features of *Clumppling* (Table 1):

**Table 1.** Features of cluster alignment methods: (1) *Clumpp*, (2) *Clumpak*, (3) *Pong*, and (4) *Clumppling*.

Feature	1	2	3	4
Aligns same-*K* replicates on the same set of individuals	✓	✓	✓	✓
Pairwise alignment uses established optimization tools	✗	✗	✓	✓
Detects modes among same-*K* replicates algorithmically	✗	✓	✓	✓
Mode detection uses established network tools	✗	✓	✗	✓
Aligns major modes of *K* and K+1	✗	✓	✓	✓
Aligns all modes of *K* and K+1	✗	✗	✗	✓
Mode alignment uses all replicates in a mode	✗	✓	✗	✓
Aligns modes of *K* and K+d, d>1	✗	✗	✗	✓

1. Like *Pong*, *Clumppling* performs pairwise alignments using established algorithms from combinatorial optimization.
2. Like *Clumpak*, *Clumppling* performs mode detection using established community detection algorithms from network theory.
3. Unlike the other methods, *Clumppling* performs alignments between all modes at successive values of *K*, not only the major modes.
4. Unlike the other methods, *Clumppling* performs alignments between replicates that differ in number of clusters by more than one.

*Clumppling* combines benefits of *Clumpak* and *Pong* in relying on ideas used for optimization and alignment in other fields. It performs alignments in the settings considered by *Clumpp*, *Clumpak*, and *Pong* and also expands to new settings.

## 3 Methods

### 3.1 Overview

In the application of mixed-membership unsupervised clustering, the first step is to obtain multiple clustering replicates at each of multiple values of the number of clusters *K*. Beginning from these replicates, the procedure of *Clumppling* to align replicates and to extract modes is as follows:

1. Group replicates according to the number of clusters *K*.
2. For each group of replicates with a shared *K*:
  - For each pair of replicates in the group, obtain an optimal alignment with minimal pairwise cost.
  - Detect subgroups of replicates belonging to shared modes.
  - Obtain the consensus membership of each mode.
3. Align pairs of modes across different values of *K* using their consensus memberships.
4. Visualize the aligned modes.

This pipeline follows that of *Clumpak* and *Pong* (*Clumpp* does not perform steps 2b, 2c, or 3, and its step analogous to 2a does not involve finding all pairwise alignments). However, *Clumpak* and *Pong* address only a special case of step 3 in which pairs of modes to be aligned across *K* values are the major modes of replicates with *K* and K+1 clusters. *Clumppling* provides alignments between each pair of modes, major and minor, considering replicates that can have numbers of clusters that differ by more than one. This new step is informative on how major and minor modes relate across *K* values and can also assist in aggregating clustering results with large *K* in which nonconsecutive *K* values may be of interest.

For steps 2a and 2b, the central steps of the alignment procedure, we seek to improve performance and run time over previous methods. First, for pairwise alignment of replicates in step 2a, we use integer linear programming (ILP) from optimization theory. Second, for community detection in step 2b, we use the Louvain algorithm from network theory.

### 3.2 Initial setup: dissimilarity between replicates

Consider two replicates from a clustering algorithm on *N* individuals. Replicate 1, with $K_{1}$ clusters, can be represented as a matrix *Q* of size $N\times K_{1}$. Entry $q_{ik}$ is the inferred membership coefficient of individual *i* in cluster *k*. Replicate 2, with $K_{2}$ clusters, is a matrix *P* of size $N\times K_{2}$. Without loss of generality, suppose $K_{1}\geq K_{2}$.

To align two replicates, we need a measure that quantifies the similarity or dissimilarity of matrices *Q* and *P*. The problem of aligning the replicates can then be formulated as a problem of maximizing the similarity, or minimizing the dissimilarity, between membership matrices, one of whose columns is rearranged according to various proposed alignments.

For $K_{1}=K_{2}=K$, *Clumpp* uses a pairwise similarity between two membership matrices, defined with the Frobenius matrix norm $||\cdot |{|}_{F}$ as

(1) $$G^{\prime }(Q,P)=1-\frac{||Q-P|{|}_{F}}{\sqrt{2N}}.$$

*Clumpp* seeks to find the optimal alignment of *R* replicates by maximizing a measure of mean pairwise similarity of the *R* replicates, termed $H^{\prime }$. *Clumpak* uses this same method for the case of $K_{1}=K_{2}=K$.

Equation (1) applies only to membership matrices of the same size. For $K_{1}>K_{2}$, we can consider measures that decompose the calculation into two levels: similarity or dissimilarity first between clusters, one from one replicate and one from the other, and second, between replicates.

*Pong* uses this two-level idea to define the similarity between replicates. Let $q_{\cdot i}$ denote the membership coefficients in cluster *i* of replicate 1, with entries $q_{\ell i}$ for ℓ=1,2,…,N; $q_{\cdot i}$ is the *i*th column of matrix *Q*. Similarly, let $p_{\cdot j}$ denote the membership coefficients in cluster *j* of replicate 2. A cluster similarity is derived from the Jaccard index on the overlap in membership coefficients between clusters $q_{\cdot i}$ and $p_{\cdot j}$ as

(2) $$J(q_{\cdot i},p_{\cdot j})=1-\sqrt{\frac{\sum_{\ell \in N^{*}}{\left(q_{\ell i}-p_{\ell j}\right)}^{2}}{2|N^{*}|}},$$

where $N^{*}=\{\ell \in \{1,2,\ldots ,N\}:q_{\ell i}+p_{\ell j}>0\}$. $N^{*}$ is the set of rows with nonzero membership in cluster *i* of *Q*, cluster *j* of *P*, or both.

The similarity between replicates is then defined as the mean cluster similarity across all clusters for a pair of replicates:

(3) $$J(Q,P)=\frac{1}{K_{1}}\sum_{i=1}^{K_{1}}J(q_{\cdot i},p_{\cdot j^{\prime }}),$$

where $j^{\prime }$ is the cluster in *P* to which cluster *i* in *Q* aligns.

If $K_{1}=K_{2}=K$ and all entries of the membership matrices are nonzero, i.e. $N^{*}=N$, then J has a form close to $G^{\prime }$, though not quite equal to it. Equations (1) and (3) can be rewritten as follows:

(4) $$\begin{array}{c} G^{\prime }(Q,P)=1-\frac{\sqrt{\sum_{k=1}^{K}\sum_{\ell =1}^{N}{\left(p_{\ell k}-q_{\ell k}\right)}^{2}}}{\sqrt{2N}}, \end{array}$$

(5) $$\begin{array}{c} J(Q,P)=1-\frac{1}{K}\frac{\sum_{k=1}^{K}\sqrt{\sum_{\ell =1}^{N}{\left(p_{\ell k}-q_{\ell k}\right)}^{2}}}{\sqrt{2N}}. \end{array}$$

$G^{\prime }$ ranges from 0 to 1. $G^{\prime }=1$ trivially if P=Q. $G^{\prime }=0$ if for all ℓ=1,2,…,N, $p_{\ell k}=1$ and $q_{\ell k^{\prime }}=1$ for some $k^{\prime }\neq k$. In this case, ${\left(p_{\ell k}-q_{\ell k}\right)}^{2}=1$ for 2 *N* of the *NK* pairs (ℓ,k).

However, the similarity measure J used by *Pong* does not reach 0. Because $\sum_{\ell =1}^{N}{\left(p_{\ell k}-q_{\ell k}\right)}^{2}\leq N$ for all k=1,2,…,K, $\sum_{k=1}^{K}\sqrt{\sum_{\ell =1}^{N}{\left(p_{\ell k}-q_{\ell k}\right)}^{2}}\leq K\sqrt{N}$. Therefore, $J(Q,P)\geq 1-K\sqrt{N}/(K\sqrt{2N})=1-1/\sqrt{2}$.

For *Clumppling*, we seek a dissimilarity measure for membership matrices that (1) permits $K_{1}>K_{2}$, and (2) spans the full unit interval [0, 1], with a value of 0 for matrices with no overlap and a value of 1 for identical memberships. We use a measure with the two-level composition of *Pong* but with a form more similar to that of *Clumpp* and *Clumpak*.

For the dissimilarity between cluster *i* of replicate 1 and cluster *j* of replicate 2, *Clumppling* uses

(6) $$C(q_{\cdot i},p_{\cdot j})=\frac{1}{2N}\sum_{\ell =1}^{N}{\left(q_{\ell i}-p_{\ell j}\right)}^{2}.$$

For the dissimilarity between two replicates with $K_{1}=K_{2}=K$, it uses

(7) $$D(Q,P)=\sum_{k=1}^{K}C(q_{\cdot k},p_{\cdot k}).$$

Continuing with $K_{1}=K_{2}$, dissimilarity D(Q,P) is related to similarity $G^{\prime }$ [Equations (1) and (4)]:

(8) $$D(Q,P)=\frac{1}{2N}\sum_{k=1}^{K}\sum_{\ell =1}^{N}{\left(q_{\ell k}-p_{\ell k}\right)}^{2}={\left[1-G^{\prime }(q_{\ell k},p_{\ell k})\right]}^{2}.$$

We will see shortly how to proceed if $K_{1}>K_{2}$.

### 3.3 Step 2a: pairwise alignment

Given a dissimilarity measure, the alignment of two replicates involves permuting the clusters of one replicate—the columns of its associated matrix—to minimize the dissimilarity with the other replicate.

If $K_{1}=K_{2}=K$, then aligning two replicates is the problem of finding the optimal one-to-one permutation that minimizes D(Q,α(P)), where α(P) is the matrix *P* with columns permuted under a permutation α of [K]={1,2,…,K}. Minimizing the dissimilarity is equivalent to maximizing the similarity, the problem considered by *Clumpp*.

If $K_{1}>K_{2}$, then the alignment between two replicates involves a many-to-one mapping from $\left[K_{1}]=\{1,2,\ldots ,K_{1}\right\}$ to $\left[K_{2}]=\{1,2,\ldots ,K_{2}\right\}$. For $K_{1}\geq K_{2}$ in general, denote the alignment by α—i.e. α(i)=j for $i\in [K_{1}]$ and $j\in [K_{2}]$. Each *i* is mapped to exactly one *j*, and each *j* is the image of at least one *i*. The dissimilarity between replicates with alignment α is a function of *Q*, *P*, and α:

(9) $$D(Q,\alpha (P))=\sum_{k=1}^{K_{1}}C(q_{\cdot k},p_{\cdot \alpha (k)}).$$

Note that if $K_{1}=K_{2}$, then we have

(10) $$\sum_{k=1}^{K_{1}}C(q_{\cdot k},p_{\cdot \alpha (k)})=\frac{1}{2N}\sum_{\ell =1}^{N}\sum_{k=1}^{K_{1}}{\left(q_{\ell k}-p_{\ell \alpha (k)}\right)}^{2}\leq 1,$$

as for each ℓ=1,2,…,N, $\sum_{k=1}^{K_{1}}{\left(q_{\ell k}-p_{\ell \alpha (k)}\right)}^{2}$ is bounded above by 2. The maximal dissimilarity satisfies $D(Q,\alpha (P))\leq \frac{1}{2N}2N=1$.

If $K_{1}>K_{2}$, however, then the alignment is no longer one-to-one, and for a specific ℓ, $\sum_{k=1}^{K_{1}}{\left(q_{\ell k}-p_{\ell \alpha (k)}\right)}^{2}$ can exceed 2. For instance, for *Q* with dimensions N×3 and *P* with dimensions N×2, an individual ℓ with $\left(q_{\ell 1},q_{\ell 2},q_{\ell 3})=(1,0,0\right)$, $\left(p_{\ell 1},p_{\ell 2})=(0,1\right)$, and alignment mapping α(1,2,3)=(1,2,2), $\sum_{k=1}^{K_{1}}{\left(q_{\ell k}-p_{\ell \alpha (k)}\right)}^{2}=3$. Hence, if $K_{1}>K_{2}$, then the maximal value of D(Q,α(P)) can exceed 1. Nevertheless, for a specific pair (Q,P), a smaller value of D(Q,α(P)) always indicates a closer alignment.

The problem of finding the optimal alignment between a pair of replicates can then be formulated as computing $\text{arg}{\text{min}}_{\alpha }D(Q,\alpha (P))$. *Clumppling* uses ILP to perform this optimization.

Linear programming (LP) concerns the problem of maximizing or minimizing a linear objective function subject to linear equality and inequality constraints (Schrijver 1998). These constraints form a feasible region of a convex polyhedron for variables that are optimized. LP problems are represented in canonical form by $\min_{x}\{c^{T}x|Ax\leq b,x\geq 0\}$, where $x={\left(x_{1},x_{2},\ldots ,x_{n}\right)}^{T}$ records the *n* variables to be optimized, $c^{T}x$ is the objective function (cost function) to be minimized, and Ax≤b and x≥0 summarize the linear constraints.

ILP problems have additional constraints that some variables are integers; in those dimensions, the feasible set for the variables is restricted to lattice points in the polyhedron. An ILP problem in which all variables must be integers can be represented in canonical form $\min_{x}\{c^{T}x|Ax\leq b,x\geq 0,x\in Z^{n}\}$. Although ILP problems are NP-complete (Schrijver 1998), we can capitalize on extensive effort devoted to solving them as standard problems in optimization theory.

To formulate the pairwise alignment problem with ILP, we place dissimilarities $C(q_{\cdot i},p_{\cdot j})$ between pairs of clusters in two replicates in a $K_{1}\times K_{2}$ matrix *C*. Denote the alignment α as a $K_{1}\times K_{2}$ indicator matrix *W*, where

$$W_{ij}=\{\begin{array}{c} 1,\;\;\text{if}\;\alpha (i)=j,\\ 0,\;\;\text{otherwise}. \end{array}$$

Because α is a many-to-one mapping, each row of *W* in constrained to sum to exactly 1, and each column of *W* has sum at least 1.

The dissimilarity between two replicates in Equation (9) can be written as follows:

(11) $$D(Q,\alpha (P))=\sum_{i=1}^{K_{1}}\sum_{j=1}^{K_{2}}W_{ij}C_{ij}.$$

The alignment problem can now be formulated with ILP:

  $$\begin{array}{ccc} \underset{W}{\text{arg }\text{min}} & \sum_{i=1}^{K_{1}}\sum_{j=1}^{K_{2}}W_{ij}C_{ij}, & \end{array}$$

(12) $$\begin{array}{ccc} \text{subject to} & \sum_{j=1}^{K_{2}}W_{ij}=1 & \;\text{for each}\;i\in [K_{1}],\\ & \sum_{i=1}^{K_{1}}W_{ij}\geq 1 & \;\text{for each}\;j\in [K_{2}],\\ & W_{ij}\in \{0,1\} & \;\text{for each}\;i\in [K_{1}],j\in [K_{2}]. \end{array}$$

In fact, this minimization is an instance of *binary linear programming* (Wolsey 2020), in which variables are restricted to zeros and ones. The canonical form of this ILP problem appears in Supplementary Methods. A pairwise alignment problem framed in this manner can then be solved by standard ILP methods. *Clumppling* uses the branch-and-cut algorithm (Mitchell 2002). The optimal solution $w^{*}$ that minimizes the objective function corresponds to the optimal alignment under the chosen dissimilarity measure [Equation (12), as reformulated in Supplementary Equation (1)].

### 3.4 Step 2b: mode detection via community detection

For all replicates with equally many clusters, an optimal alignment is obtained for each pair using ILP as described in the previous section. Suppose there are $R_{K}$ replicates with *K* clusters, and their membership matrices are $Q_{K}^{\left(1\right)},Q_{K}^{\left(2\right)},\ldots ,Q_{K}^{\left(R_{K}\right)}$. Between a pair of replicates *i* and *j*, the optimal alignment of $Q_{K}^{\left(i\right)}$ to $Q_{K}^{\left(j\right)}$ is $\alpha_{ij}^{*}$, extracted from the solution $w^{*}$ to Equation (12) that gives the minimal dissimilarity $D_{\alpha_{ij}^{*}}(Q_{K}^{\left(i\right)},Q_{K}^{\left(j\right)})$.

To align all replicates simultaneously, *Clumppling* constructs an undirected graph $G_{K}=(V,E)$ using replicates as nodes $V=\{1,2,\ldots ,R_{K}\}$. Edge set $E=\{(i,j)|i=1,2,\ldots ,R_{K},j=1,2,\ldots ,R_{K}\}$ has weights $u_{ij}$ negatively weighted by the normalized dissimilarity of optimal alignments: $u_{ij}=1$ for i=j, and for i≠j,

(13) $$u_{ij}=\frac{D_{\text{max}}-D_{\alpha_{ij}^{*}}(Q_{K}^{\left(i\right)},Q_{K}^{\left(j\right)})}{D_{\text{max}}-D_{\text{min}}},$$

where $D_{\text{min}}=\min_{i\neq j}\{D_{\alpha_{ij}^{*}}(Q_{K}^{\left(i\right)},Q_{K}^{\left(j\right)})\}$ and $D_{\text{max}}=\max_{i\neq j}\{D_{\alpha_{ij}^{*}}(Q_{K}^{\left(i\right)},Q_{K}^{\left(j\right)})\}$. A higher weight indicates greater similarity of two replicates under their optimal alignment. The $R_{K}\times R_{K}$ matrix of edge weights is symmetric.

We seek to find sets of replicates that are collectively similar to one another when optimally aligned, or *modes*. For this task, we rely on community detection algorithms (Fortunato 2010, Javed *et al.* 2018). In a graph, communities are groups of nodes that are more densely connected within the group than outside the group. A community in the graph $G_{K}$ corresponds to a mode in the unsupervised clustering analysis. In terms of the associated edge-weight matrix, a matrix with nontrivial communities has a block-diagonal structure, with two or more blocks corresponding to communities. Entries within a block tend to exceed entries outside it for the associated rows and columns—indicating greater edge weights for node pairs within the same community than for pairs not in the same community.

*Clumppling* first tests a null hypothesis of no community structure. We use a test of Tokuda (2018), based on differences between (i) random symmetric matrices with a block-diagonal community structure in which within-block off-diagonal entries are distributed differently from outside-block off-diagonal entries, and (ii) random symmetric matrices in which all off-diagonal entries are independently and identically distributed. Considering the largest and smallest eigenvalues of two transformed versions of the symmetric edge-weight matrix of the graph, the null hypothesis is rejected if one or both eigenvalues (of either matrix) lies outside specified intervals. In our application, if the null hypothesis of no community structure is rejected at p=0.01, then *Clumppling* proceeds to identify community structure in the undirected weighted graph $G_{K}$.

For community detection, *Clumpak* uses Markov clustering (MCL) (Van Dongen 2000), employing a threshold to remove some edges with lower weights from the network—i.e. to replace smaller edge weights with values of 0—thereby reducing the density of edges. *Clumppling* instead uses the Louvain method (Blondel *et al.* 2008), which does not require premodification of the network. This algorithm has a “resolution” parameter that affects the size of the detected communities; larger values typically find smaller communities and more of them. *Clumppling* allows the user to specify its value, with a default of 1.

The outcome of mode detection via community detection algorithms is a set of communities of nodes, each of which corresponds to a subset of replicates that belong to the same mode. Modes are disjoint so that replicate each belongs to exactly one mode. Suppose $m_{K}$ communities are detected, which we denote $M_{K}^{1}$ through $M_{K}^{m_{K}}$, with $M_{K}^{i}\cap M_{K}^{j}=\emptyset$ for all distinct $i,j\in [m_{k}]$, $\cup_{\ell =1}^{m_{k}}M_{K}^{\ell }=V$, and $\sum_{\ell =1}^{m_{k}}|M_{K}^{\ell }|=R_{K}$. It is possible for a mode to possess only one replicate, $|M_{K}^{\ell }|=1$, although this “singleton” situation is somewhat unusual.

### 3.5 Step 2c: consensus memberships for modes

For each mode ℓ, we obtain a consensus membership matrix in one of two ways. First, we obtain a *mean* membership matrix of its replicates:

(14) $${\bar{Q}}_{K}^{\ell }=\frac{1}{\left|M_{K}^{\ell }\right|}\sum_{i\in M_{K}^{\ell }}Q_{K}^{\left(i\right)},$$

where, for simplicity, the $Q_{K}^{\left(i\right)}$ are treated as having already been aligned.

We also obtain a *representative* membership matrix, the matrix of the replicate that has the largest sum of edge weights within the community:

(15) $${\tilde{Q}}_{K}^{\ell }=\underset{Q_{K}^{\left(i\right)}:i\in M_{K}^{\ell }}{\text{arg }\text{max}}\sum_{j\in M_{K}^{\ell },j\neq i}u_{ij}.$$

Either ${\bar{Q}}_{K}^{\ell }$ or ${\tilde{Q}}_{K}^{\ell }$ can be used as the consensus membership of replicates in the mode. *Clumppling* uses ${\bar{Q}}_{K}^{\ell }$ as its default.

Suppose the set of the distinct numbers of clusters is K. After building networks of replicates for each *K*, we obtain a set of modes together with consensus memberships:

(16) $${\left\{\left(M_{K}^{\ell },{\bar{Q}}_{K}^{\ell }):\ell \in [m_{K}\right]\right\}}_{K\in K}.$$

### 3.6 Step 3: alignment of modes across *K*

Finally, with modes defined, we align modes across values of *K*. In particular, we order the values of K∈K in decreasing order: $K_{1}\geq K_{2}\geq \cdots \geq K_{\left|K\right|}$. We then obtain a multipartite graph of the pairwise alignments between modes across different values of *K*.

For adjacent *K* values in K, $K_{i}$ and $K_{j}$, where j=i+1, there exist $m_{K_{i}}\times m_{K_{j}}$ pairs of modes. For each such pair, we use ILP [Equation (12)] to align consensus memberships from Equation (16). The optimal dissimilarity is $D({\bar{Q}}_{K_{i}}^{\ell_{i}},\alpha^{*}({\bar{Q}}_{K_{j}}^{\ell_{j}}))$, where $\ell_{i}\in [m_{K_{i}}]$, $\ell_{j}\in [m_{K_{j}}]$, and $\alpha^{*}$ is the mapping of clusters of ${\bar{Q}}_{K_{j}}^{\ell_{j}}$ to clusters of ${\bar{Q}}_{K_{i}}^{\ell_{i}}$ that produces the optimum.

We then use these dissimilarities between modes as weights to create a bipartite graph between modes of $K_{i}$ and modes of $K_{j}$. For instance, the weight between mode $\ell_{i}$ of $K_{i}$ and mode $\ell_{j}$ of $K_{j}$ can be set to

(17) $$\mu_{\left(K_{i},\ell_{i}),(K_{j},\ell_{j}\right)}=1-D({\bar{Q}}_{K_{i}}^{\ell_{i}},\alpha^{*}({\bar{Q}}_{K_{j}}^{\ell_{j}})),$$

where larger weights indicate closer alignments. Note that theoretically, the dissimilarity *D* can exceed 1 for a pair of modes with different numbers of clusters. However, such a situation requires pairs of clusters to be matched extremely poorly between different values of *K*, an unlikely scenario after optimal alignment. Hence, negative weights in Equation (17) are unlikely.

Combining the bipartite graphs between pairs of adjacent values of *K*, we obtain a |K|-partite graph representing alignments across modes with different numbers of clusters. We call our approach to the alignment of modes across *K* the “direct” approach.

For consecutive values of *K*, we also consider a second approach that modifies the method of *Pong*; we term this second approach the “merge” approach. This approach enumerates all possible ways to merge a pair of clusters from the mode with K+1 clusters to produce *K* clusters. It then uses our ILP method to align two matrices of the same size. A total of $\left(\begin{array}{c} K+1\\ 2 \end{array}\right)$ alignments are performed, and the one that achieves the smallest dissimilarity is chosen to be the optimal alignment between the two modes. Note that unlike the “direct” approach, which permits nonconsecutive *K* values, the “merge” approach requires the values of *K* to be consecutive.

### 3.7 Visualization

To display all modes at all numbers of clusters, we proceed sequentially in the order $\left(K_{1},K_{2},\ldots ,K_{\left|K\right|}\right)$, arranging values of *K* with $K_{1}\geq K_{2}\geq \ldots \geq K_{\left|K\right|}$. Proceeding from i=1 to i=|K|−1, for each adjacent pair $\left(K_{i},K_{i+1}\right)$ (e.g. K+1 and *K* if their numbers of clusters are consecutive), we choose the most closely aligned pair of modes between them as “anchors.” We then perform the following three steps:

1. All other modes with $K_{i}$ clusters are aligned to the $K_{i}$ anchor.
2. All other modes with $K_{i+1}$ clusters are aligned to the $K_{i+1}$ anchor.
3. The modes of these two different *K* values are then aligned according to the alignment of the $\left(K_{i},K_{i+1}\right)$ anchor pair.

For example, consider three numbers of clusters $\left(K_{1},K_{2},K_{3}\right)$, each with two modes. Suppose the most closely aligned mode pair for $\left(K_{1},K_{2}\right)$ is ($K_{1}$-Mode_1_, $K_{2}$-Mode_2_) and that for $\left(K_{2},K_{3}\right)$, it is ($K_{2}$-Mode_1_, $K_{3}$-Mode_2_). First, we align modes of $\left(K_{1},K_{2}\right)$: (1) $K_{1}$-Mode_2_ is aligned to the anchor $K_{1}$-Mode_1_. (2) $K_{2}$-Mode_1_ is aligned to the anchor $K_{2}$-Mode_2_. (3) Clusters in modes of these two *K* values are rearranged according to the alignment between $K_{1}$-Mode_1_ and $K_{2}$-Mode_2_. Next, we align modes of $\left(K_{2},K_{3}\right)$ in the same way: (1) $K_{2}$-Mode_2_ is aligned to the anchor $K_{2}$-Mode_1_—which was already accomplished in the previous step (1), as alignments between modes with the same *K* apply symmetrically. (2) $K_{3}$-Mode_1_ is aligned to the anchor $K_{3}$-Mode_2_. (3) Across modes of $K_{2}$ and $K_{3}$, the alignment follows that between $K_{2}$-Mode_1_ and $K_{3}$-Mode_2_. In this way, all modes across $\left(K_{1},K_{2},K_{3}\right)$ are aligned.

For visualization of aligned modes, *Clumppling* plots each mode as a “classic” structure plot—a stacked bar chart of equal height—with clusters represented by different colors (e.g. Rosenberg 2004). The number of replicates in a mode is marked above the associated plot. To visualize relationships between modes with different numbers of clusters, modes appear in a multipartite graph according to the across-*K* alignment. Modes with the same *K* appear in the same “layer,” where the number of layers is K, the number of distinct *K* values considered. For each *K*, modes are ordered in decreasing size (i.e. the number of replicates in the mode) and decreasing within-mode similarity [described in Equation (18)]. Modes with adjacent *K* values are joined by edges colored based on the edge weight [Equation (17)], with darker colors indicating larger weights and closer alignments. Minimal dissimilarities between pairs of modes under their optimal alignment—i.e. the optimal objective function values from Equation (12)—appear as labels on the edges, with smaller values indicating closer alignments. To visualize the variability within a mode, in addition to the structure plot, *Clumppling* provides a histogram of pairwise dissimilarities under optimal alignment of replicates within modes (Supplementary Fig. S1).

## 4 Evaluation of performance

The *Clumppling* implementation is described in the Supplementary Methods. We compare the alignment performance of *Clumppling* to the two existing methods, *Clumpak* and *Pong*, that align replicates across *K* values. Because they only align equal or consecutive *K* values, the evaluation does so as well—although *Clumppling* accommodates replicates with numbers of clusters differing by more than 1.

### 4.1 Performance measure

For replicates with a shared *K*, we use a performance measure based on the similarity score $H^{\prime }$ of *Clumpp* and *Clumpak*. For a mode $M_{K}^{\ell }$, let $H_{\ell }^{\prime }$ denote the mean similarity score for all pairs of replicates in the mode after its replicates $Q_{1},Q_{2},\ldots Q_{\left|M_{K}^{\ell }\right|}$ are all aligned pairwise:

(18) $$H_{\ell }^{\prime }=\{\begin{array}{cc} 1, & |M_{K}^{\ell }|=1,\\ \frac{1}{\left(\begin{array}{c} \left|M_{K}^{\ell }\right|\\ 2 \end{array}\right)}\sum_{i=1}^{|M_{K}^{\ell }|-1}\sum_{j=i+1}^{\left|M_{K}^{\ell }\right|}G^{\prime }(Q_{i},\alpha^{*}(Q_{j})), & |M_{K}^{\ell }|>1, \end{array}$$

where $\alpha^{*}$ is interpreted as the mapping of clusters of $Q_{j}$ to clusters of $Q_{i}$ for the optimal alignment of $Q_{i}$ and $Q_{j}$.

Next, to obtain a mean similarity score involving all replicates and modes, we calculate a weighted similarity score $\bar{H}$ that assigns each replicate the mean similarity score of its associated mode:

(19) $${\bar{H}}_{K}=\frac{1}{R_{K}}\sum_{\ell =1}^{m_{K}}|M_{K}^{\ell }|H_{\ell }^{\prime }.$$

We modify Equation (19) to exclude singleton modes ℓ with $|M_{K}^{\ell }|=1$. Because singletons always have similarity score 1 in Equation (18), their existence can upwardly bias the weighted similarity score. With $s_{K}$ singleton modes, the singleton-excluded weighted similarity score becomes

(20) $${\tilde{H}}_{K}=\frac{1}{R_{K}-s_{K}}\sum_{\ell =1,\;|M_{K}^{\ell }|\neq 1}^{m_{K}}|M_{K}^{\ell }|H_{\ell }^{\prime }.$$

Note that removal of the singletons reduces the number of replicates that need to be aligned; it is useful to track the value $s_{K}$ along with ${\tilde{H}}_{K}$.

Because we compare the performance of *Clumppling* with methods that only support the alignment of replicates with consecutive values of *K*, we use a performance measure suited to consecutive *K* values. To evaluate across-*K* alignments, we measure the $G^{\prime }$ similarity [Equation (1)] between the most closely aligned pair of modes aligned across each (K,K+1). For this computation, an optimal alignment $\alpha^{*}$ has first already been identified for this pair of modes in Section 3.6. Two of the clusters in the mode with K+1 clusters are necessarily matched to the same cluster in the mode with *K* clusters, or $\alpha^{*}(i)=\alpha^{*}(j)$ for exactly one unordered pair of i,j∈[K+1], i≠j. We apply the alignment $\alpha^{*}$: we merge these two clusters, adding their columns in the membership matrix together to produce a single column. Mathematically, suppose the original N×(K+1) membership matrix for the mode with K+1 clusters is *P*, with columns ${\left\{p_{k}\right\}}_{k\in [K+1]}$. It now becomes a new N×K membership matrix $P^{\prime }$ with columns

$$p_{k}^{\prime }=\sum_{i:i\in [K+1],\alpha^{*}(i)=k}p_{i},\;\text{for}\;k=1,2,\ldots ,K.$$

Now the two membership matrices, *Q* and $P^{\prime }$, both have size N×K. $G^{\prime }(Q,P^{\prime })$ is computed for these two matrices.

Note that because this similarity calculation merges clusters, it measures the similarity of a mode with *K* clusters and a mode with K+1 clusters according to its value for a quantity optimized by the “merge” and not the “direct” approach. Hence, it is expected to have higher values when the “merge” rather than the “direct” approach is used to produce the optimal alignment. Because *Pong* uses the “merge” approach to align modes with consecutive numbers of clusters, the calculation evaluates *Clumppling* against *Pong* by a measure that *Pong* seeks to optimize.

We evaluate the performances of all four possible combinations of the mode consensus approach and the approach for performing alignments. That is, we use either “representative” [Equation (14)] or “average” [Equation (15)] as the consensus membership of a mode. For alignments of modes with *K* and K+1 clusters, we use either the “direct” approach or the “merge” approach in identifying the alignment with the minimal dissimilarity [Equation (9)].

### 4.2 Datasets

We demonstrate the use of *Clumppling* and compare the alignment performance of the methods with two datasets. The first contains an unsupervised *Admixture* analysis of 399 individuals focused on the human population of Cape Verde. This dataset has replicates with small values of *K*. It provides an example in which many individuals, including 44 individuals in the admixed population of Cape Verde (Verdu *et al.* 2017), possess nontrivial memberships in multiple clusters; the original analysis of Verdu *et al.* (2017) considered 50 replicates each at K=2,3,4,5; we reanalyzed those replicates. For this dataset, we ran *Clumppling* using the default resolution parameter of 1 for the Louvain mode detection.

The second dataset provides an example of alignment with relatively large values of *K*: a study of 600 chickens from 20 populations (Rosenberg *et al.* 2001) focused on values of K=17,18,19, and we add K=20,21 here (“chicken dataset”). We begin from the original data, 27 genotyped loci in each of the 600 individuals, running *Structure* (Pritchard *et al.* 2000) for 20 replicates for each *K* from 17 to 21. We ran *Structure* with a burn-in period of length 5000 in the “Admixture” model followed by 50000 MCMC repetitions, as in Rosenberg *et al.* (2001). For mode detection in *Clumppling*, we used 1.05 for the resolution parameter; for this more challenging dataset, increasing the resolution above the default of 1 leads to a larger number of modes but with greater within-mode similarity.

### 4.3 Analysis of clustering replicates

**Figure 1.**
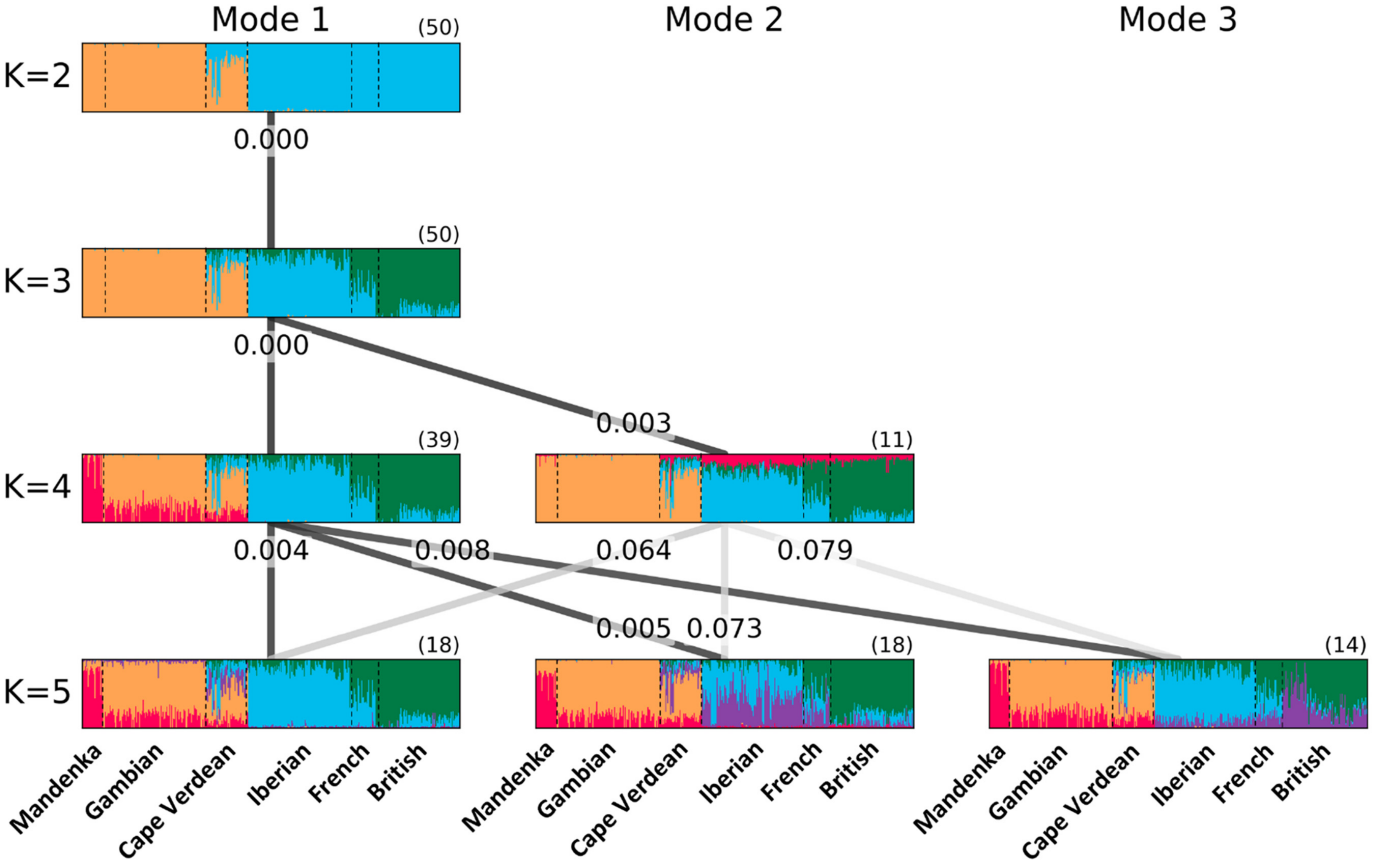
*Clumppling*-aligned modes for the Cape Verde dataset (*K* from 2 to 5), using the mean memberships as mode consensus and the “direct” approach to alignment across *K* values. The multipartite graph shows the alignment across different *K*. Edges are colored by the edge weight [Equation (17)]; darker color indicates a larger weight and thus better alignment. The numbers on the edges are the optimal solutions for pairwise alignments, representing minimum values in Equation (12). Each structure plot displays a mode, where the modes for the same *K* appear in decreasing order by their size—marked in parentheses above the top right corner of each plot—and then their within-mode similarity (if there is a tie in size).

For each value of *K*, we used *Clumppling* to align the 50 *Admixture* replicates for the Cape Verde dataset. Alignments based on mean memberships for the mode consensus and the “direct” approach for alignment across *K* values appear in Fig. 1.

**Figure 2.**
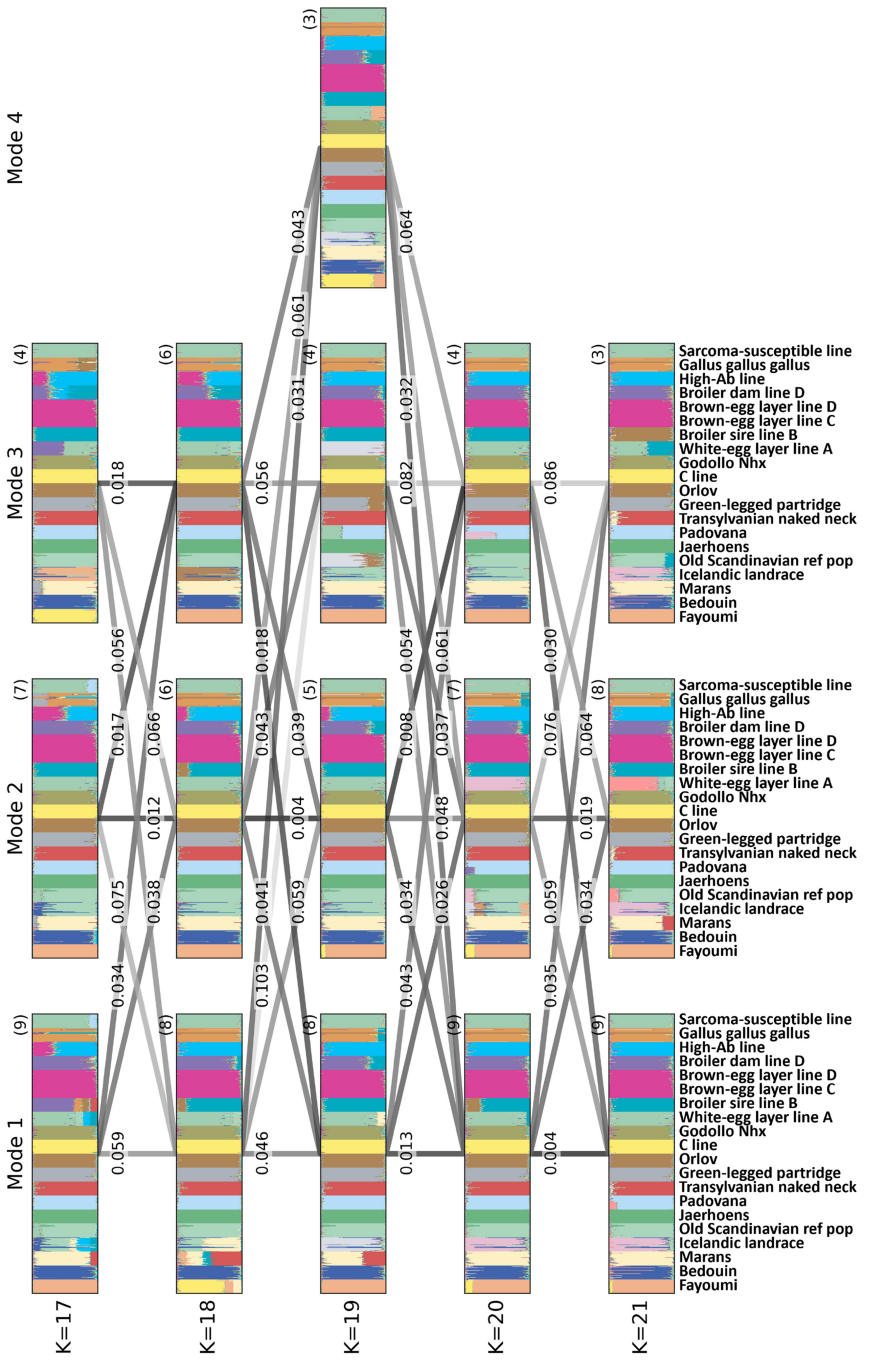
*Clumppling*-aligned modes for the chicken dataset (*K* from 17 to 21), using the mean memberships as mode consensus and the “direct” approach to alignment across *K* values. The figure design follows Figure 1.

For the chicken dataset, for each *K*, alignments based on mean memberships for the mode consensus and the “direct” approach for alignment across *K* values appear in Fig. 2. An additional analysis of alignments across non-consecutive *K* values appears in Supplementary Fig. S2.

For comparison, we ran *Clumpak* and *Pong*. *Clumpak* uses the *LargeKGreedy* algorithm of *Clumpp* to align replicates for fixed *K* values. In the MCL algorithm, it uses a threshold automatically generated from the graph properties to control inclusion of edges of the graph. It uses the *Distruct for many K’s* feature to align single results—major modes—for different *K* values. *Clumpak* alignment results appear in Supplementary Fig. S3.

We ran *Pong* (Behr *et al.* 2016) using its sum-squared distance metric for calculating the similarity between clusters; we chose the sum-squared distance rather than the Jaccard similarity in *Pong*, as it is closer to the objective used by *Clumppling*. A fixed threshold of 0.9 is chosen for the Cape Verde data and 0.955 for the chicken data to exclude edges with weights below the threshold from the pairwise similarity graph of replicates for mode detection. We chose these values to be lower than the *Pong* default of 0.97 in order to avoid producing large numbers of singleton modes. When identifying disjoint cliques in the graph of pairwise similarities for mode detection, we used the *Pong* default greedy approach to iteratively remove the maximal clique from the graph if no disjoint cliques are found. Alignment results for *Pong* appear in Supplementary Figs S4 and S5.

For within-*K* alignments, the performance of *Clumppling*, *Clumpak*, and *Pong* appears for Cape Verde in Supplementary Table S1; Supplementary Table S2 shows the numbers of replicates detected within modes by the three methods for the two datasets. Supplementary Tables S3 and S4 show corresponding results for the chicken dataset. For between-*K* alignments, *Clumppling* is evaluated in each of four combinations: using representative or average memberships, and using the “merge” and “direct” approaches to alignment. The performance of *Clumppling*, *Clumpak*, and *Pong* in across-*K* alignments for the Cape Verde dataset appears in Supplementary Table S5 and for the chicken dataset in Supplementary Table S6.

### 4.4 Performance for within-*K* alignment

In the Cape Verde dataset, for K=2 and K=3, all three methods find a single mode (Supplementary Table S2). No singletons are observed, and the singleton-excluded weighted similarity score $\tilde{H}$ is 1 for all three methods (Supplementary Table S1). For K=4, although *Clumpak* gives the largest score, this score discards 11 singletons. For K=5, *Clumppling* has the fewest modes and the largest within-mode similarity between replicates.

In the chicken dataset, at different values of *K*, *Clumppling* achieves consistently greater values of the singleton-excluded similarity score (Supplementary Table S3). It does so while finding no singleton modes; the other two methods both identify singletons at most values of *K* (Supplementary Table S4).

### 4.5 Performance for across*-K* alignment

In the Cape Verde dataset, across-*K* alignments have comparable performance for the various methods, with high similarity scores (Supplementary Table S5). In the more challenging chicken dataset, with larger values of *K*, *Clumppling* produces the largest similarity scores between the most closely aligned pair of modes at consecutive values of *K* (Supplementary Table S6). The choice of the “representative” approach for mode consensus and the “merge” approach for cluster alignment gives the highest scores, but the three other choices all produce large values for the score as well. *Clumpak* and *Pong* achieve comparably high scores only in one of four transitions between *K* values (20–21 for *Clumpak*, 17–18 for *Pong*).

### 4.6 Run time

Run time with *Clumppling* (using the “direct” approach) is comparable to *Pong*; both are faster than *Clumpak*. In *Clumppling*, the “direct” approach is faster than the “merge” approach. A detailed comparison of the run time for the three methods appears in Supplementary Results and Supplementary Table S7.

## 5 Discussion

*Clumppling* is a new method for aligning replicate mixed-membership unsupervised clustering analyses. Building upon *Clumpp* (Jakobsson and Rosenberg 2007), *Clumpak* (Kopelman *et al.* 2015), and *Pong* (Behr *et al.* 2016), it performs alignment tasks that have not been addressed by earlier methods—alignment of all modes for one value of *K* with all modes of another value of *K*, and alignment of modes across nonconsecutive *K* values. *Clumppling* applies algorithms from combinatorial optimization and network theory in producing similar alignments to those obtained by the other methods (Figs 1 and 2 and Supplementary Figs S3–S5), often with higher values of a similarity score for replicates within modes at fixed *K* (Supplementary Tables S1 and S3) or modes at consecutive *K* values (Supplementary Tables S5 and S6), and in comparable or reduced computation time (Supplementary Table S7). Thus, it compares favorably with other methods in terms of its novel features, algorithmic justification, performance measures, and run time.

### 5.1 Algorithmic innovations

*Clumppling* introduces methodological advances for cluster alignment. Though *Pong* previously described the alignment problem as a standard combinatorial optimization problem, the *Clumppling* ILP formulation of the alignment of two replicates allows it to capitalize on efficiencies of ILP solvers. Hence, *Clumppling* is comparable in speed to *Pong*.

The ILP formulation also enables *Clumppling* to address new scenarios not covered by *Pong*. In particular, the many-to-one matching that it permits for clustering replicates with different values of the number of clusters makes it possible for *Clumppling* to perform alignments across values of *K*, including across nonconsecutive values. Such alignments can be useful, e.g. in analyses for which *K* extends over a large range (Funk *et al.* 2020); for large datasets in which computation is slow, it may be sensible to perform exploratory clustering with select values of *K*—say, every fifth value—to then summarize with *Clumppling*, and only then, if necessary, to consider consecutive *K* in a meaningful range.

Finally, combining the advance from *Pong* in formulating cluster alignment in terms of a classic setting in optimization with the advance from *Clumpak* of using community detection algorithms, *Clumppling* is able to perform a more comprehensive analysis of all observed modes. In particular, *Clumppling* aligns all modes across *K* values, rather than aligning only single modes at each *K* value, as in *Clumpak* and *Pong*. This alignment is informative to clarify clustering patterns in scenarios in which multimodality arises as *K* is increased but a major mode reappears as *K* is increased still further (e.g. Fig. 7 of Wang *et al.* 2007).

The formalization of the cluster alignment problem here establishes a framework for further enhancement. By clarifying the components of the problem—pairwise alignment of replicates at fixed *K* (step 2a), mode detection at fixed *K* (step 2b), defining the consensus of modes (step 2c), and aligning modes across *K* (step 3)—each component can be separately investigated. Optimization methods other than the ILP branch-and-cut algorithm and network-based clustering methods other than the Louvain algorithm can be further tested for improvements in their associated steps.

### 5.2 Empirical performance

The performance differences in our empirical examples are relatively small. In the Cape Verde example, alignments were clear across the methods, all of which performed comparably (Supplementary Tables S1, S2, and S5). In our more difficult chicken example, *Clumppling* produced the highest value for the mean similarity of replicates within modes (Supplementary Table S3), identified the fewest singletons (Supplementary Table S4), and produced the highest similarity scores between modes with consecutive values of *K* (Supplementary Table S6). In both cases, the modes themselves are similar across methods (Fig. 1 and Supplementary Figs S3A, S4A, and S5A for Cape Verde, and Fig. 2 and Supplementary Figs S3B, S4B, and S5B for chickens).

Together, *Clumpp*, *Clumpak*, and *Pong* have been widely used, all performing well in typical empirical settings. When clustering algorithms uncover clear structure—e.g. with replicable co-clustering of some individuals in one cluster and other individuals in another—the proper alignment is often clear, and it is likely to be found by all methods. In a modeling study describing alignment cost under a Dirichlet model, we have found that a correct permutation often has cost far below that of the other permutations (Liu *et al.* 2022). *Clumppling* can be added to the list of methods that can be used to find this permutation.

### 5.3 Methodological choices and extensions

In developing *Clumppling*, we have made decisions about a number of methodological trade-offs. For the pairwise alignment step, *Pong* previously used the polynomial-time Hungarian algorithm for alignments at a fixed value of *K*. In *Clumppling*, we have chosen to use ILP, which is not polynomial-time and can be slower than the approach of *Pong*, though still faster than *Clumpak* (Supplementary Table S7). However, ILP offers the ability to facilitate alignments across both consecutive and nonconsecutive values of *K*; *Pong* accommodates only consecutive values.

For the alignment cost function, our framework allows any dissimilarity measure between replicates as the objective for the ILP problem—provided that it is a linear combination of pairwise between-cluster dissimilarities. Our specific quadratic function of entries in two membership matrices is grounded in the analysis of Liu *et al.* (2023). In particular, if two replicates have the same number of clusters, then the dissimilarity that *Clumppling* seeks to minimize via ILP is exactly (a constant multiple of) the alignment cost from Liu *et al.* (2023).

Choices in community detection affect the granularity of the modes obtained. In one extreme, each replicate is its own mode; in the other, replicates with truly distinct co-clustering patterns are grouped in the same mode. *Clumppling* follows *Clumpak* in using an adaptable parameter for tuning this granularity. Nevertheless, it is possible that replicates visually distinguishable as belonging to distinct modes might be grouped. Such patterns can sometimes be diagnosed by the appearance of membership vectors that, within individual replicates, are near a permutation of (1,0,0,…,0), but that are not near a simplex vertex in the mean of replicates within the mode. In applications, users can increment the “resolution” parameter, e.g. by 0.05 or 0.01, choosing larger values to increase granularity and smaller values to decrease it.

### 5.4 Conclusions

With its formulation of the cluster alignment problem in defined steps, combination of pairwise alignment ideas based on *Pong* and community detection based on *Clumpak*, and addition of new features for mode alignment across values of *K*, *Clumppling* can assist in the many cluster alignments that take place in population-genetic data analysis. Notably, however, mixed-membership clustering, also sometimes known as soft or fuzzy clustering, has broad applications beyond population genetics, including elsewhere in bioinformatics, as well as in image analysis, marketing, and text mining (De Oliveira and Pedrycz 2007, Airoldi *et al.* 2014). Problems of comparing and visualizing multiple clustering results in a general context have perhaps been of greater interest for hard clustering (Meilă 2007, Zhou *et al.* 2009, L’Yi *et al.* 2015), in which memberships of individuals are assigned to single clusters. For similar problems of soft clustering, the methods from population genetics—*Clumppling* and its predecessors—are available. *Clumppling* can be applied to any membership-based clustering algorithms applied multiple times on the same set of entities, and it potentially has broad applications in diverse uses of mixed-membership cluster analysis.

## Supplementary Material

btad751_Supplementary_Data

## Data Availability

The software is available as a python package at https://github.com/PopGenClustering/Clumppling. The data underlying this article are available in its online supplementary material.
