# Supplementary material for: Clumppling: cluster matching and permutation program with integer linear programming
Source: Bioinformatics. 2023 Dec 14;40(1):btad751. doi: 10.1093/bioinformatics/btad751 (PMC10766593; doi:10.1093/bioinformatics/btad751)
Supplement: btad751_Supplementary_Data [file btad751_supplementary_data.zip › Supplementary Materials.pdf]

Supplementary Materials

Supplementary Tables

K	*Clumpak*		*Pong*		*Clumppling*	
	m (s)	$\tilde{H}$	m (s)	$\tilde{H}$	m (s)	$\tilde{H}$
2	1 (0)	**1.000**	1 (0)	**1.000**	1 (0)	**1.000**
3	1 (0)	**1.000**	1 (0)	**1.000**	1 (0)	**1.000**
4	12 (11)	**0.979**	4 (1)	0.942	2 (0)	0.938
5	6 (4)	0.808	7 (2)	0.811	3 (0)	**0.818**

Table S1: Performance of *Clumpak*, *Pong*, and *Clumppling* in aligning replicates at fixed values of K for the Cape Verde dataset. For each of the four values of K, 50 *Admixture* replicates are considered. m, number of modes; s, number of singleton modes; $\tilde{H}$, singleton-excluded weighted similarity score (eq. 20). The highest value(s) of each row is bolded.

K	*Clumpak*	*Pong*	*Clumppling*
2	50	50	50
3	50	50	50
4	39	41, 5, 3	39, 11
5	31, 15	21, 9, 9, 7, 2	18, 18, 14

Table S2: Sizes of detected modes for the Cape Verde dataset. The first entry corresponds to the number of replicates in the major mode, among 50 total replicates for each K from 2 to 5. Mode sizes are listed in decreasing order. Singleton modes are omitted.

K	*Clumpak*		*Pong*		*Clumppling*	
	m (s)	$\tilde{H}$	m (s)	$\tilde{H}$	m (s)	$\tilde{H}$
17	2 (1)	0.630	8 (3)	0.689	3 (0)	**0.709**
18	3 (0)	0.755	5 (3)	0.753	3 (0)	**0.788**
19	3 (1)	0.752	4 (1)	0.722	4 (0)	**0.803**
20	4 (2)	0.811	2 (0)	0.773	3 (0)	**0.834**
21	5 (2)	0.870	1 (0)	0.800	3 (0)	**0.877**

Table S3: Performance of *Clumpak*, *Pong*, and *Clumppling* in aligning replicates at fixed values of K for the chicken dataset. For each of the five values of K, 20 *Structure* replicates are considered. The table design follows Table S1.

K	*Clumpak*	*Pong*	*Clumppling*
17	19	6, 4, 3, 2, 2	9, 6, 5
18	15, 3, 2	12, 5	7, 7, 6
19	16, 3	15, 2, 2	8, 5, 4, 3
20	14, 4	17, 3	9, 7, 4
21	13, 3, 2	20	9, 8, 3

Table S4: Sizes of detected modes for the chicken dataset. The table design follows Table S2.

K	*Clumpak*	*Pong*	*Clumppling* (representative)		*Clumppling* (average)	
			merge	direct	merge	direct
(2,3)	0.994	0.994	0.994	0.994	0.994	0.994
(3,4)	**0.998**	**0.998**	**0.998**	0.944	**0.998**	0.949
(4,5)	0.938	0.900	0.950	0.877	**0.950**	0.935

Table S5: Performance of *Clumpak*, *Pong*, and *Clumppling* in aligning replicates at consecutive values of K for the Cape Verde dataset. The G' similarity (eq. 1) between the most closely aligned pair of modes is shown for each $(K, K+1)$. *Clumppling* performance is measured using each of four choices (representative or average memberships for the consensus of a mode, merge or direct approach for alignment across K values). The highest value(s) of each row is bolded.

K	*Clumpak*	*Pong*	*Clumppling* (representative)		*Clumppling* (average)	
			merge	direct	merge	direct
(17,18)	0.831	0.955	**0.980**	0.894	0.900	0.900
(18,19)	0.761	0.780	**0.977**	0.959	0.940	0.935
(19,20)	0.882	0.788	**0.979**	**0.979**	0.943	0.908
(20,21)	0.903	0.791	**0.979**	0.958	0.944	0.939

Table S6: Performance of *Clumpak*, *Pong*, and *Clumppling* in aligning replicates at consecutive values of K for the chicken dataset. The table design follows Table S5.

Data	Task	*Clumpak*	*Pong*	*Clumppling* (representative)		*Clumppling* (average)	
				merge	direct	merge	direct
Cape Verde	within-K	6m34s	-	16.58s	16.93s	16.95s	17.00s
	across-K	2s	-	0.30s	0.07s	0.30s	0.07s
	total	6m36s	36.62s	20.74s	20.94s	20.94s	21.91s
Chicken	within-K	43m38s	-	6.19s	6.12s	6.14s	6.10s
	across-K	8s	-	37.10s	0.45s	36.73s	0.44s
	total	43m46s	16.71s	47.72s	10.95s	47.25s	10.94s

Table S7: Run-time comparison of *Clumpak*, *Pong*, and *Clumppling*. The within-K time is the time for all steps involved in aligning replicates within-K and detecting modes among them—for all K values. The across-K time is the time for all steps involved in aligning modes across-K for all pairs of adjacent K values. For *Clumppling*, this step includes alignment of all modes between the adjacent K values. For *Clumpak* and *Pong*, it only includes the alignment of major modes. For *Pong*, only a total time is available.

Supplementary Figures

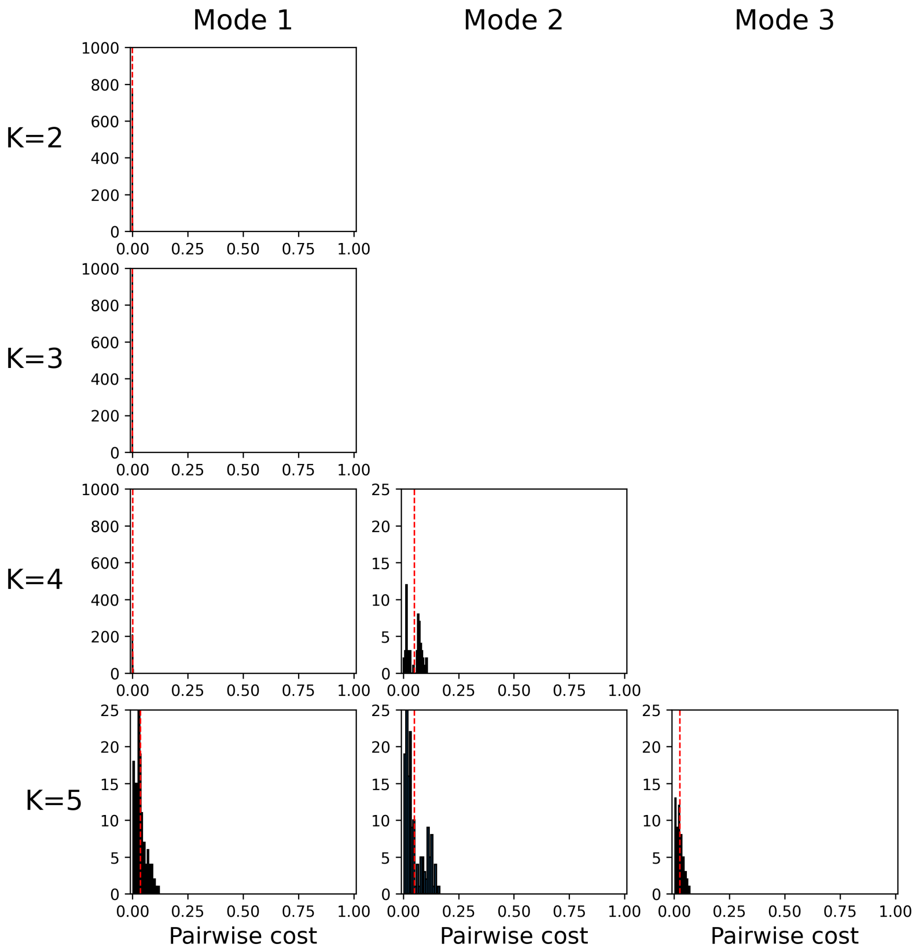

Figure S1: Distribution of pairwise dissimilarities between replicates within each mode under optimal alignment, for *Clumppling*-aligned modes of the Cape Verde dataset. The modes are the same as those displayed in Figure 1 and are arranged in the same way. For each mode, the plot shows a histogram of pairwise dissimilarities between all pairs of replicates within that mode, each computed as the optimal value of the objective function in eq. 12. The mean dissimilarity is marked by the red dashed line.

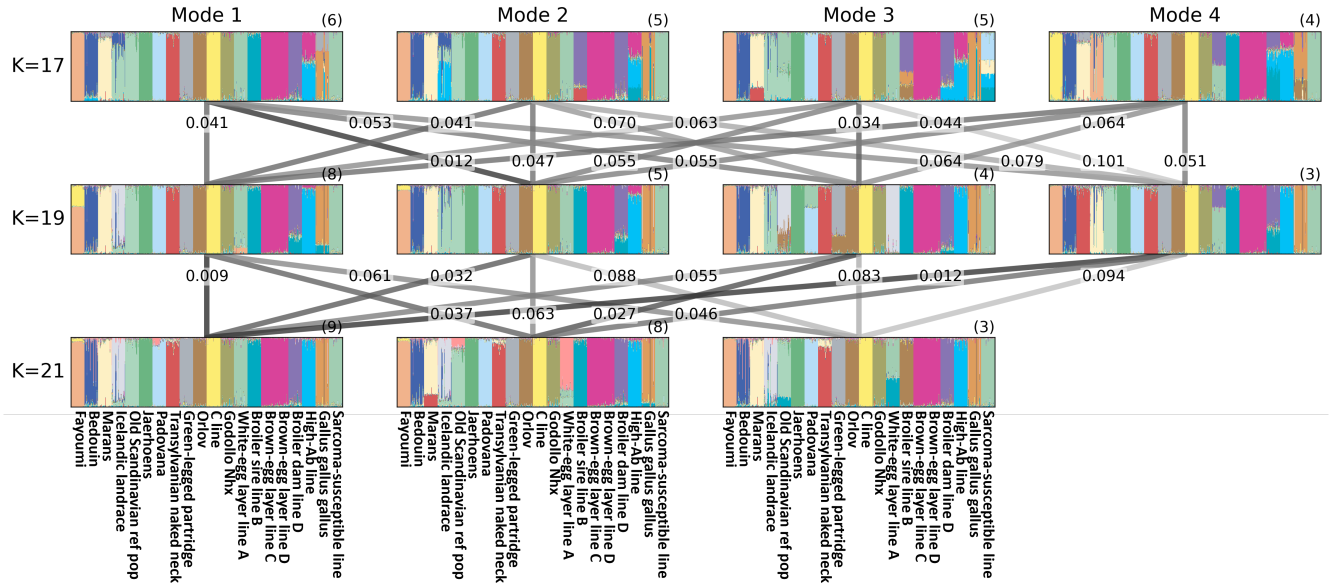

Figure S2: *Clumppling*-aligned modes for the chicken dataset with non-consecutive numbers of clusters ($K = 17$, 19, and 21), using the mean memberships as mode consensus and the "direct" approach to alignment across K values. Note that with non-consecutive K values, the "merge" strategy does not apply. The figure design follows Figures 1 and 2.

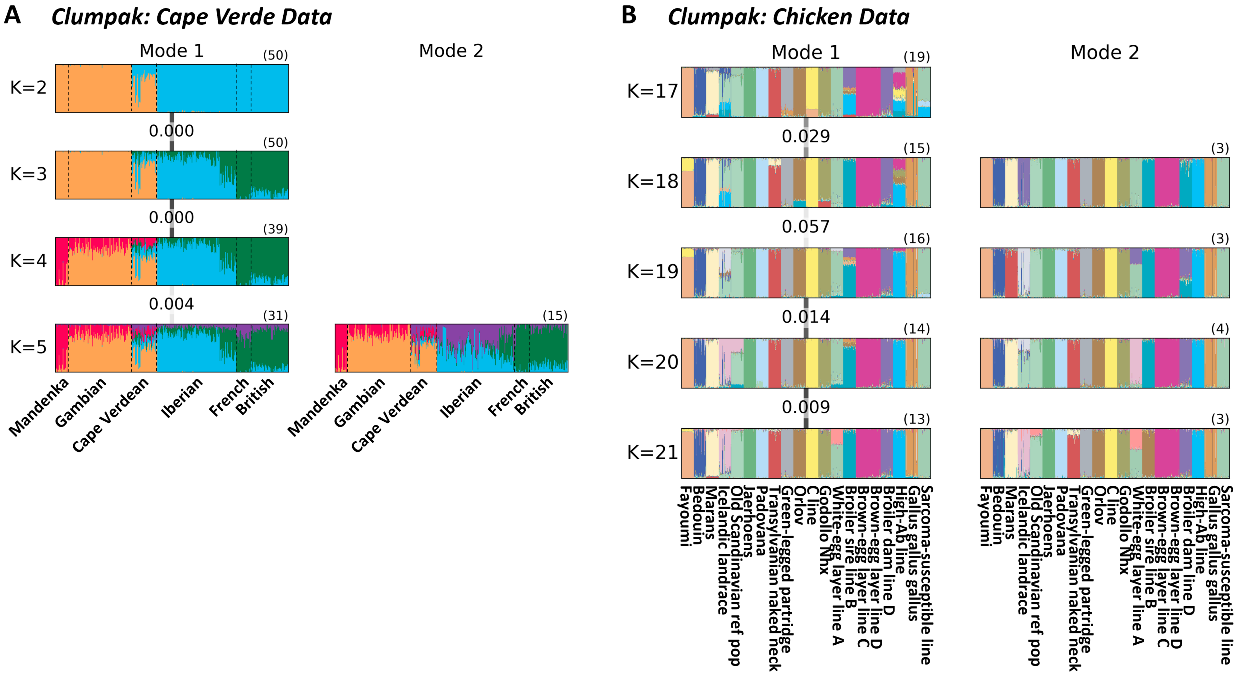

Figure S3: *Clumpak*-aligned modes. *Clumpak* uses the mean memberships across all replicates in a mode as the consensus memberships of the mode. (A) Cape Verde dataset. (B) Chicken dataset. The figure design follows Figures 1 and 2, except that edges appear only between the major modes for adjacent K values.

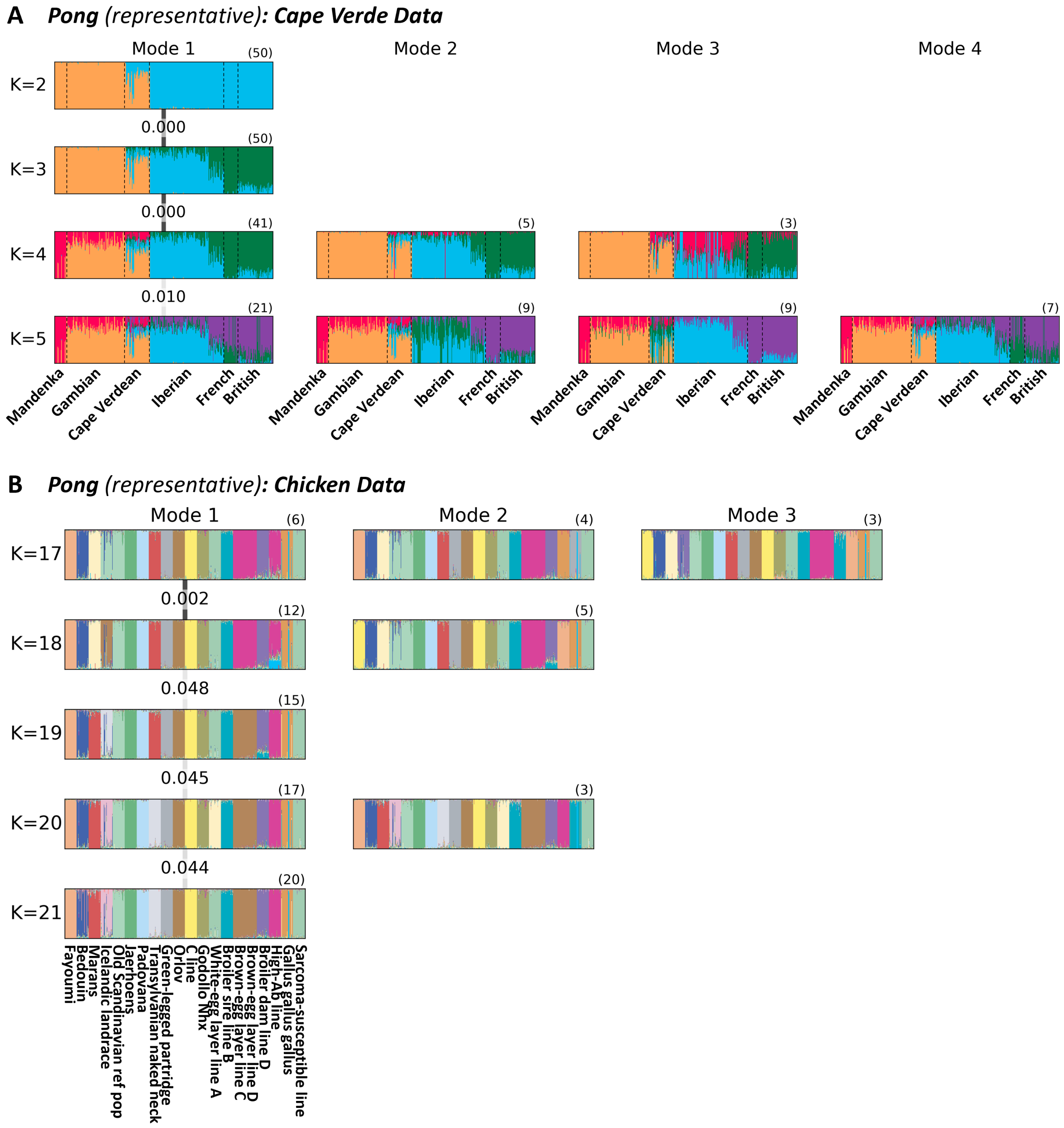

Figure S4: *Pong*-aligned modes. *Pong* uses a representative replicate from each mode as the consensus memberships of the mode. (A) Cape Verde dataset. (B) Chicken dataset, where modes are plotted using the representative replicate. The figure design follows Figures 1 and 2, except that edges appear only between the major modes for adjacent K values.

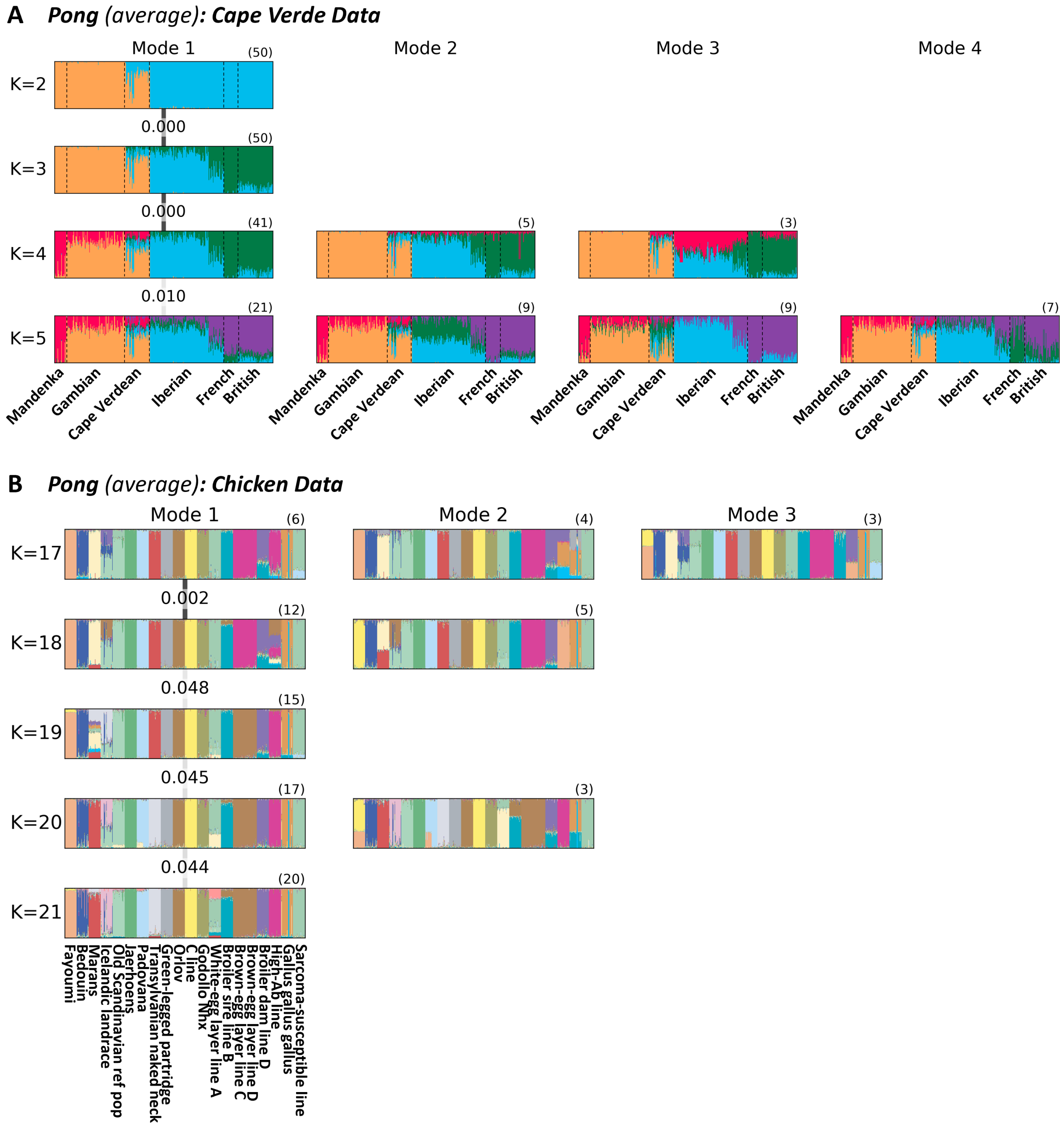

Figure S5: *Pong*-aligned modes, with modes plotted using mean memberships as mode consensus. This approach is not the consensus membership used by *Pong* for across-K alignment, but it is shown here to facilitate direct comparison to *Clumppling* (Figures 1 and 2) and *Clumpak* (Figure S3). (A) Cape Verde dataset. (B) Chicken dataset. The figure design follows Figures 1 and 2, except that edges appear only between the major modes for adjacent K values.

Supplementary Methods: ILP

The canonical form for use in general solvers of integer and binary linear programming problems is [Papadimitriou and Steiglitz, 1998, p. 307]

$$\begin{aligned} \min_{\mathbf{x}} \quad & \mathbf{c}^T\mathbf{x}, \\ \text{subject to} \quad & A\mathbf{x} \ \leq \mathbf{b}, \\ & x_j \quad \text{integer (or binary)} \qquad \text{(for some or all } j). \end{aligned}$$

We rewrite the problem in eq. 12 in this canonical form. To do this, we first need to arrange matrix W into a vector $\mathbf{w}$. We reshape the two-dimensional W matrix into a one-dimensional vector $\mathbf{w}$ of length K_1K_2 proceeding through the rows in row-major order. We similarly reshape the C matrix into a vector $\mathbf{c}$ of length K_1K_2. That is,

$$\begin{aligned} \mathbf{w}_{(i-1)K_2+j} &= W_{ij} \ \text{ for } i \in [K_1], j \in [K_2], \\ \mathbf{c}_{(i-1)K_2+j} &= C_{ij} \ \text{ for } i \in [K_1], j \in [K_2]. \end{aligned}$$

We have thus obtained the objective function $\mathbf{c}^T\mathbf{w}$ in canonical form.

It remains rewrite the constraints in the form $A\mathbf{w} \leq \mathbf{b}$. We encode the constraints in eq. 12 in a matrix A of size $L \times K_1K_2$ and a vector $\mathbf{b}$ of length $L = 2K_1 + K_2$. Eq. 12 has two sets of constraints. The first set is the equality constraints $\{\sum_{j=1}^{K_2} W_{ij} = 1\}_{i\in[K_1]}$, each of which is equivalent to two *inequality* constraints $\sum_{j=1}^{K_2} W_{ij} \leq 1$ and $\sum_{j=1}^{K_2} W_{ij} \geq 1$. The latter is rewritten as $-\sum_{j=1}^{K_2} W_{ij} \leq -1$ to fit the $A\mathbf{w} \leq b$ format. These constraints are encoded by two sets of entries in A and $\mathbf{b}$. For $\ell = 1, 2, \ldots, K_1$, $\mathbf{b}_\ell = 1$, and

$$A_{\ell m} = \begin{cases} 1, & \text{if } (\ell-1)K_2 + 1 \leq m \leq \ell K_2, \\ 0, & \text{otherwise.} \end{cases}$$

For $\ell = K_1+1, K_1+2, \ldots, 2K_1$, $\mathbf{b}_\ell = -1$, and

$$A_{\ell m} = \begin{cases} -1, & \text{if } (\ell - K_1 - 1)K_2 + 1 \leq m \leq (\ell - K_1)K_2, \\ 0, & \text{otherwise.} \end{cases}$$

The next constraints in eq. 12 are the inequality constraints $\{\sum_{i=1}^{K_1} W_{ij} \geq 1\}_{j\in[K_2]}$. Each constraint is rewritten $-\sum_{i=1}^{K_1} W_{ij} \leq -1$. Hence, for $\ell = 2K_1+1, 2K_1+2, \ldots, 2K_1+K_2$, $\mathbf{b}_\ell = -1$, and

$$A_{\ell m} = \begin{cases} -1, & \text{if } m \in \{\ell - 2K_1 + nK_2\}_{n=0,1,\ldots,K_1-1}, \\ 0, & \text{otherwise.} \end{cases}$$

The first $2K_1$ rows of the inequality $A\mathbf{w} \leq \mathbf{b}$ ensure the equality constraints and the last K_2 rows ensure the inequality constraints. The integer constraints in eq. 12 need no transformation. With all the constraints transformed into canonical form, eq. 12 can be rewritten

$$\begin{aligned} \arg\min_{\mathbf{w}} \quad & \mathbf{c}^T\mathbf{w}, \\ \text{subject to} \quad & A\mathbf{w} \leq \mathbf{b}, \\ & \mathbf{w} \in \{0,1\}^n. \end{aligned} \tag{1}$$

This canonical form of the ILP problem can then be directly inserted into integer linear programming solvers, including the *GLPK_MI* solver [Makhorin, 2008] that is used by *Clumppling*.

Supplementary Methods: Implementation

Clumppling is implemented in Python. For ease of use, *Clumppling* supports input formats from multiple clustering methods, including *Structure*, *Admixture*, and *fastStructure*. We use the mixed-integer solver *GLPK_MI* [Makhorin, 2008] provided through the `CVXPY` package [Agrawal et al., 2018, Diamond and Boyd, 2016]. This solver uses the branch-and-cut algorithm to efficiently solve mixed-integer problems, a class of problems that includes ILP problems as a subset.

The Louvain community detection method is implemented in the Python `community` API. The multi-partite graph visualization is performed through the `NetworkX` Python package [Hagberg

et al., 2008].

The program requires an input path for data files, an output path to store results, and a parameter specifying the format of the input files. It offers various optional inputs, including (1) the "resolution" parameter for mode detection, as discussed in Section 3.4; (2) a choice of "representative" replicates or "average" memberships for mode representation; (3) a choice of the "merge" or "direct" approach for merging pairs of clusters when aligning replicates with $K+1$ and K clusters. Additional decisions concern features of the visualization.

Supplementary Results: Run Time

We compare the run time of *Clumppling* to *Clumpak* and *Pong*. In particular, we evaluate execution time for the demonstration datasets we used in performance evaluation (Section 4). The same machine with CPU clock speed 3.7 GHz was used to execute all three programs.

Calculation of the run time

The run time is recorded separately for two tasks during the execution: (i) within-K alignment and mode detection for all K values, which are performed together ("within-K" for short), and (ii) across-K alignment for all pairs of adjacent K values ("across-K"). *Clumppling* records both times.

The run time for *Clumpak* is computed from the timestamps in its output log file. The time for steps in alignment "within-K" and "across-K" are obtained separately by subtracting the start timestamp from the end timestamp of the series of steps corresponding to each task.

Pong outputs the time its algorithms take for characterization of modes and alignment of Q matrices as "match time" and "align time." These times are not equivalent to our "within-K" and "across-K" tasks, and for a better comparison to *Clumppling*, we obtain the total command-line execution time of *Pong* (with the interactive visualization disabled). Similarly, we obtain the total execution time of *Clumppling* program without visualizations. We term this quantity the "total" time. Note that the total time for *Clumppling* includes some additional time to load and process files, so it is slightly larger than the sum of the within-K and across-K times. For *Clumpak*, we

sum the time for the two tasks, within-K and across-K, to be its total time; this total does not include the step of generating the summary file with visualizations. The data processing time is already included in the time for the two tasks.

Comparison of methods

For the chicken dataset with large K, computation time is noticeably different among methods (Table S7). *Clumppling* and *Pong* are faster than the *Clumpak* default greedy algorithm. Among the different approaches for running *Clumppling*, the "merge" approach is slower than the "direct" approach.

For the Cape Verde dataset with small K, the run time difference is not as large, but we can still observe that *Clumppling* and *Pong* are computationally more efficient than *Clumpak*.

References

Akshay Agrawal, Robin Verschueren, Steven Diamond, and Stephen Boyd. A rewriting system for convex optimization problems. *Journal of Control and Decision*, 5(1):42–60, 2018.

Steven Diamond and Stephen Boyd. CVXPY: A Python-embedded modeling language for convex optimization. *Journal of Machine Learning Research*, 17(83):1–5, 2016.

Aric Hagberg, Pieter Swart, and Daniel Schult. Exploring network structure, dynamics, and function using NetworkX. Technical report, Los Alamos National Laboratory, Los Alamos, NM, 2008.

Andrew Makhorin. GLPK (GNU linear programming kit). *http://www.gnu.org/s/glpk/glpk.html*, 2008.

Christos H Papadimitriou and Kenneth Steiglitz. *Combinatorial Optimization: Algorithms and Complexity*. Dover, Mineola, NY, 1998.
