# Supplementary material for: Clumppling: cluster matching and permutation program with integer linear programming
Source: Bioinformatics. 2023 Dec 14;40(1):btad751. doi: 10.1093/bioinformatics/btad751 (PMC10766593; doi:10.1093/bioinformatics/btad751)
Supplement: btad751_Supplementary_Data [file btad751_supplementary_data.zip › Clumppling_Code.zip!/Clumppling_Manual.pdf]

Clumppling Manual

V.0.2.0

Xiran Liu
xiranliu@stanford.edu

Nov 28, 2023

Contents

1 Introduction **2**

2 Quick Start **2**

3 Download and Installation **3**
3.1 Open a Command-Line Interpreter . . . 3
3.2 Install Python 3 . . . 4
3.3 Install Conda and Create Virtual Environment . . . 5
3.3.1 Install `conda` . . . 5
3.3.2 Create a virtual environment . . . 5
3.4 Install *Clumppling* . . . 5
3.4.1 Install directly . . . 5
3.4.2 Install after downloading . . . 6
3.5 Check usage . . . 7

4 Running *Clumppling* **7**
4.1 Usage of the `merge_cls` parameter . . . 9
4.2 Usage of the `cd_param` parameter . . . 9

5 Input Data Format **9**
5.1 Clustering from *Structure* . . . 9
5.2 Clustering from *Admixture* . . . 10
5.3 Clustering from *fastStructure* . . . 10
5.4 General Membership Matrices from Any Clustering . . . 10

6 Output Files **10**
6.1 The "input" folder . . . 11
6.2 The "alignment_withinK" folder . . . 11
6.3 The "modes" folder . . . 11
6.4 The "alignment_across_K" folder . . . 12
6.5 The "modes_aligned" folder . . . 12
6.6 The "visualization" folder . . . 12

7 Examples **14**
7.1 Datasets . . . 14
7.1.1 Human genotype data . . . 14
7.1.2 Chicken microsatellite data . . . 15
7.2 Putting the data files under the correct directory . . . 15
7.3 Running `clumppling` on Cape Verde data . . . 15
7.4 Running `clumppling` on chicken data . . . 15
7.5 Running `clumppling` on outputs with a non-consecutive number of clusters . . . 17

8 Additional Functionalities **17**
8.1 `clumppling.diffModel`: aligning memberships of the same individuals from different models 17
8.1.1 How to run `clumppling.diffModel` 17
8.1.2 Outputs 18
8.1.3 Example 18
8.2 `clumppling.diffInd`: aligning memberships of different individuals from (possibly different) models 19
8.2.1 How to run `clumppling.diffInd` 20
8.2.2 Outputs 21
8.2.3 Example 21

9 Customizable Algorithms **22**
9.1 Custom community detection method 23
9.2 Alternative p-value for testing the existence of community structure 23

1 Introduction

Clumppling is a method for aligning multiple clustering replicates of population structure analysis, with an application specifically for multiple results of inferred ancestries from population structure analysis. It adopts integer linear programming strategies to align a pair of mixed-membership clustering replicates with either the same or a different number of clusters. It identifies modes from all replicates with the same number of clusters using community detection. It aggregates multiple clustering results by summarizing them into modes with different numbers of clusters. It also presents the results in a multipartite graph of structure plots for easy visualization.

2 Quick Start

If you are **familiar with Python and command line tools**, simply follow the steps given in this section to get a quick start on using *Clumppling*. If you are not familiar with these, **detailed instructions are provided starting in Section 3.**

1. Install Python 3 of version 3.8 and up. Make sure it is callable via `python` from the command-line shell. You may check this by `python --version` to see if the expected Python 3 version is being used.

2. Install `conda` and create a virtual environment.

```
conda create -n clumppling-env python
```

Activate the created virtual environment.

```
conda activate clumppling-env
```

3. Install the *Clumppling* package in **one of the following two ways**:

   (a) Directly install the package by

```
pip install clumppling
# Alternatively, you may install by:
# pip install https://github.com/PopGenClustering/Clumppling/archive/
  master.zip
# Or, if you have Git installed, you may also do:
# pip install git+https://github.com/PopGenClustering/Clumppling
```

   then download the example files and scripts from `https://github.com/PopGenClustering/Clumppling/tree/master/input` and `https://github.com/PopGenClustering/Clumppling/tree/master/scripts`.

   (b) Download the package files from `https://github.com/PopGenClustering/Clumppling`, then navigate to its home directory titled "Clumppling" (you may need to change the automatically generated name "Clumppling-master" to "Clumppling"), and run the command

```
pip install -e .
```

4. To see the usage of the program with a list of required and optional parameters, run

```
python -m clumppling -h
```

5. Run the program with default parameters via the command

```
python -m clumppling -i INPUT_PATH -o OUTPUT_PATH -f INPUT_FORMAT
```

where `INPUT_PATH` is the directory of all input files (clustering results), `OUTPUT_PATH` is a directory that does not exist and will be created to save the output files, and `INPUT_FORMAT` is the format of input, chosen from *structure*, *admixture*, *fastStructure* and *generalQ*.

3 Download and Installation

3.1 Open a Command-Line Interpreter

Open a command-line interpreter (i.e., a shell/command prompt/terminal).

Linux and macOS Use the built-in Terminal.

Windows You will need to get a terminal of your choice. *The default Command Prompt (available via running the* `cmd` *program) is not recommended unless you are comfortable with setting up environment path variables and are familiar with syntax differences between Command Prompt and Terminal.*

Some choices for the terminal are:

- (Recommended) Anaconda Prompt, a built-in tool from the Anaconda Navigator after you follow the instructions in Section 3.2 to install Python and those in Section 3.3.1 to install Conda using the installers, this terminal will automatically become available. Simply type "Anaconda Prompt" in the search box of the start menu and click on the popped-up application to open the command-line window.

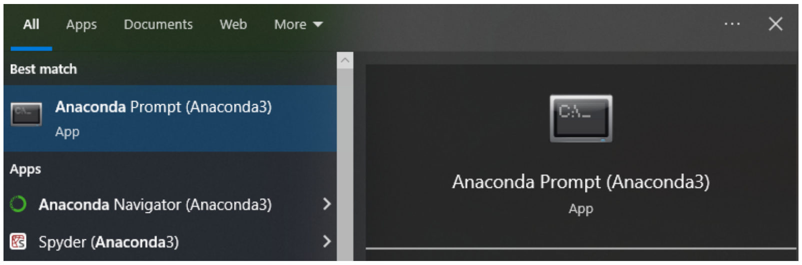

Figure 1: How to open Anaconda Prompt from Windows Start Menu search box after you install Conda.

- The built-in (for Windows 10) Windows PowerShell. Type "Windows PowerShell" in the search box of the start menu, then the application will pop up.
- Git Bash, a built-in tool available from Git after you download and run its installer from `https://git-scm.com/download/win`. Type "Git Bash" in the search box of the start menu, then the application will pop up.

Some commonly used commands Here are some useful commands that are commonly used

- `pwd` - print the absolute path name of your current working directory (*Note*: for Windows users, you will need to run `cd` instead)
- `cd DIRECTORY` - change your working directory to the given directory `DIRECTORY`

- `cd ..` - change your working directory to the parent of the current working directory
- `ls` - list the contents of the current directory (*Note*: for Windows users, you will need to run `dir` instead)
- `python` - run the Python interpreter (after you have installed Python, see Section 3.2)

3.2 Install Python 3

Clumppling is based on Python 3, so you first need to have Python 3 installed on your system.

Version Python version of at least 3.8 is recommended. The package has been tested under Python 3.8, 3.9, 3.10, and 3.11.

Linux Linux users can install Python 3 from the command window using the package manager. Make sure that you have root user access for the installation.
For RHEL (Red Hat Enterprise Linux) and CentOS users, you can install via

```
sudo yum install -y python3
```

For Ubuntu and Debian users, use

```
sudo apt-get install python3
```

MacOS and Windows MacOS and Windows users can download Python 3 from `https://www.python.org/downloads/`. Follow the links and get the corresponding installer for your system (e.g., "Windows installer (64-bit)" for Windows and "macOS 64-bit universal2 installer" for MacOS), then double-click on the installer file and follow the instructions to install Python 3. Do not forget to check the box "Add Python to environment variables" during the installation if prompted.

[Optional] Check Python version If you already have Python installed on your machine, you can check the version of your Python via the command

```
python --version
```

Note: On some systems with both Python 2 and Python 3 installed, command `python` starts the interactive Python 2 interpreter and `python3` starts the interactive Python 3. However, for convenience, we will only be using `python` prompt for the rest of the manual. If your system uses `Python 3`, you can either switch all `python` commands in this manual to `python3` and switch all `pip` commands to `pip3`, or set the alias by running

```
alias python=python3
```

[Optional] Check Python is working in the interpreter You can open a Python interpreter by typing

```
python -i
```

You will see information about the current Python version, and your cursor should appear after `>>>`. To exit the interpreter, type `exit()` after `>>>`. *Note:* There may be issues when using certain shells on Windows, like GitBash.

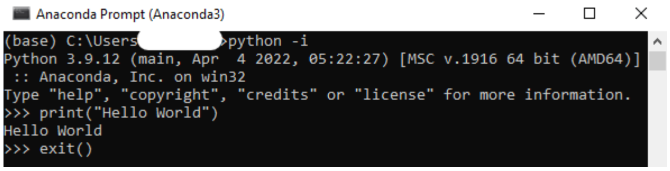

Figure 2: How to open and exit Python interpreter.

The solution is to run `winpty python -i` instead, or to set the alias by `python=winpty`.

3.3 Install Conda and Create Virtual Environment

We highly recommend creating a virtual environment using **Conda**, which will help manage package installations and avoid conflicts with your other Python projects.

3.3.1 Install `conda`

You can download the installer for Anaconda at `https://www.anaconda.com/download` then follow the instruction to install it. For Windows users, once you installed Anaconda, you will be able to use Anaconda Prompt as the command line interpreter.

You may check your Conda version by

```
conda -V
```

3.3.2 Create a virtual environment

You may refer to the official document (`https://conda.io/projects/conda/en/latest/user-guide/tasks/manage-environments.html`) for more information on the environment management with conda. Here we show how to create a virtual environment named `clumppling-env` for running *Clumppling*.

To see a list of all of your environments, run

```
conda info --envs
```

Create a virtual environment named `clumppling-env` with Python by

```
conda create -n clumppling-env python
```

Press `y` to proceed when prompted. You may use your preferred name instead of `clumppling-env` for the virtual environment. You may also specify a Python version by substituting `python=3.8` for `python` in the above line.

To activate the environment, run

```
conda activate clumppling-env # for conda 4.6 and later versions
source activate clumppling-env # for conda versions prior to 4.6
```

If you are prompted to run `conda init`, follow the instructions.

All the installation and program-running should happen in this virtual environment, i.e., steps in Section 3.4 and 4.

Once you are done using the program, deactivate the environment:

```
conda deactivate
```

To remove the environment permanently, run

```
conda remove -n clumppling-env --all
```

3.4 Install *Clumppling*

There are two ways to install the package.

The first is to install the package from the GitHub repository directly, then download the example files and scripts if you want to try the demo in Section 7.

The second is to download all package files to the local machine from GitHub, then install the package locally.

3.4.1 Install directly

Install To install *Clumppling* directly, run:

```
pip install clumppling
```

Alternatively, you may install via the following command

```
pip install https://github.com/PopGenClustering/Clumppling/archive/master.
    zip
```

Or, if you have Git installed on your system, you may run

```
pip install git+https://github.com/PopGenClustering/Clumppling
```

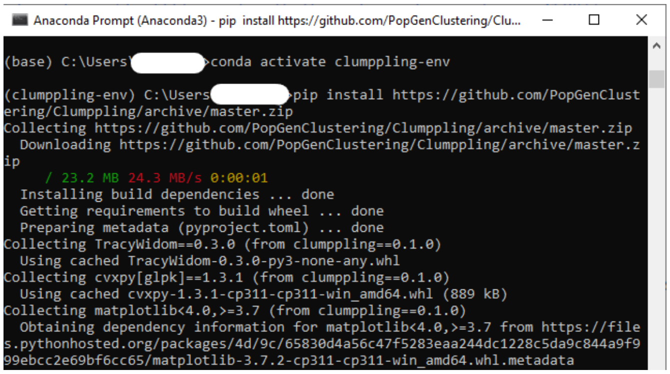

Figure 3: Activate the virtual environment and install the package.

Download Because direct installation would not automatically download the example files and scripts, you will need to get them separately from the folder "input" and "scripts" under `https://github.com/PopGenClustering/Clumppling`.

3.4.2 Install after downloading

You can also install the package after downloading the files to the local machine.

Download Download the entire directory by clicking the link `https://github.com/PopGenClustering/Clumppling/archive/refs/heads/master.zip`. Unzipping the zipped file on your local machine, you should see a folder named "Clumppling-master", which contains the "README.md" file. Rename this directory "Clumppling". Note that this renaming step is recommended for the sake of notation consistency across this manual. For example, if you download and unzip the folder to `C:/Users/USERNAME`, then after unzipping and renaming, the *Clumppling* files will show up under `C:/Users/USERNAME/Clumppling`.

Alternatively, you may clone the package files from GitHub using Git. To install Git, download the installer and follow the instructions from `https://git-scm.com/downloads`. Next, navigate to the local directory where you would like to put the files. You can then clone the repository by

```
git clone https://github.com/PopGenClustering/Clumppling.git
```

Under your current directory, a folder named "Clumppling" will show up, which contains all the files of the package. For instance, if you are cloning the files under the directory `C:/Users/USERNAME`, then after cloning, the *Clumppling* directory will show up at `C:/Users/USERNAME/Clumppling`.

Install Next, navigate to the directory named "Clumppling" where you see the files including "README.md" and "pyproject.toml". For example, if your directory for *Clumppling* is `C:/Users/USERNAME/Clumppling`, navigate into it by

```
cd C:/Users/USERNAME/Clumppling
```

Then run the following command to install the package:

```
pip install -e .
```

On your console, if you see the text ending with "successfully installed" followed by a list of packages that are newly installed, including `clumppling-0.1.0`, then this means that your installation has succeeded.

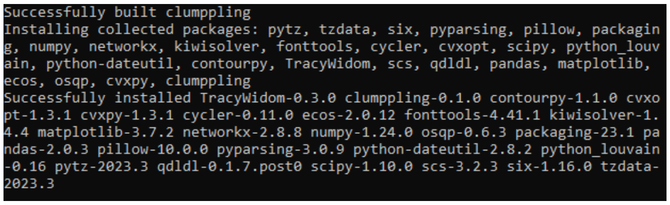

```
Successfully built clumppling
Installing collected packages: pytz, tzdata, six, pyparsing, pillow, packagin
g, numpy, networkx, kiwisolver, fonttools, cycler, cvxopt, scipy, python_louv
ain, python-dateutil, contourpy, TracyWidom, scs, qdldl, pandas, matplotlib,
ecos, osqp, cvxpy, clumppling
Successfully installed TracyWidom-0.3.0 clumppling-0.1.0 contourpy-1.1.0 cvxo
pt-1.3.1 cvxpy-1.3.1 cycler-0.11.0 ecos-2.0.12 fonttools-4.41.1 kiwisolver-1.
4.4 matplotlib-3.7.2 networkx-2.8.8 numpy-1.24.0 osqp-0.6.3 packaging-23.1 pa
ndas-2.0.3 pillow-10.0.0 pyparsing-3.0.9 python-dateutil-2.8.2 python_louvain
-0.16 pytz-2023.3 qdldl-0.1.7.post0 scipy-1.10.0 scs-3.2.3 six-1.16.0 tzdata-
2023.3
```

Figure 4: Successful installation.

[Optional] Debug If you get an Error saying that "A pyproject.toml file was found, but editable mode currently requires a setuptools-based build", then update your pip first by

```
python -m pip install --upgrade pip
```

then retry the installation.

If are any compatibility issues, which are unlikely to happen if you are using a virtual environment, please resolve them first and then go back to the step in Section 3.4 and retry the installation .

3.5 Check usage

You should have installed the package now. If you're not sure where the package is installed, you can run

```
pip show clumppling
```

This will show you the installation location as well as basic information about the package.

To check the success of the installation, you may run the following command to see the help message for the main module `clumppling`:

```
python -m clumppling -h
```

If the package has been installed successfully, you will see the following output in your command window

4 Running *Clumppling*

The main module of the package runs the alignment algorithm on clustering outputs of the same model on the same set of individuals, namely, it runs the alignment on different membership matrices $Q : N \times K$ with the same number of rows N, but not necessarily the same number of columns K. This function takes in three required arguments and several optional ones:

- `input_path` (`-i`, required): path to the folder that contains the input files, i.e. the clustering results.
- `output_path` (`-o`, required): path to a folder that the user wants to save the alignment results to, where the folder must not exist yet.
- `input_format` (`-f`, required): the input data format, chosen from `structure`, `fastStructure`, `admixture`, and `generalQ`.
- `vis` (`-v`, optional): whether to generate visualization: 0 for no, 1 for yes (default).
- `cd_param` (optional): a numerical value of the resolution parameter for the community detection Louvain algorithm (default 1.0); a larger value typically results in smaller communities and more of them, and correspondingly more and smaller modes.
- `use_rep` (optional): whether to use representative replicate as mode consensus: 0 for no (default), 1 for yes.
- `merge_cls` (optional): whether to merge all pairs of clusters to align $K + 1$ and K: 0 for no (default), 1 for yes.
- `cd_default` (optional): whether to use the default Louvain algorithm for community detection: 0 for no, 1 for yes (default). If no, then extra steps need to be taken to add a custom community detection function in the source code (see Section 9).

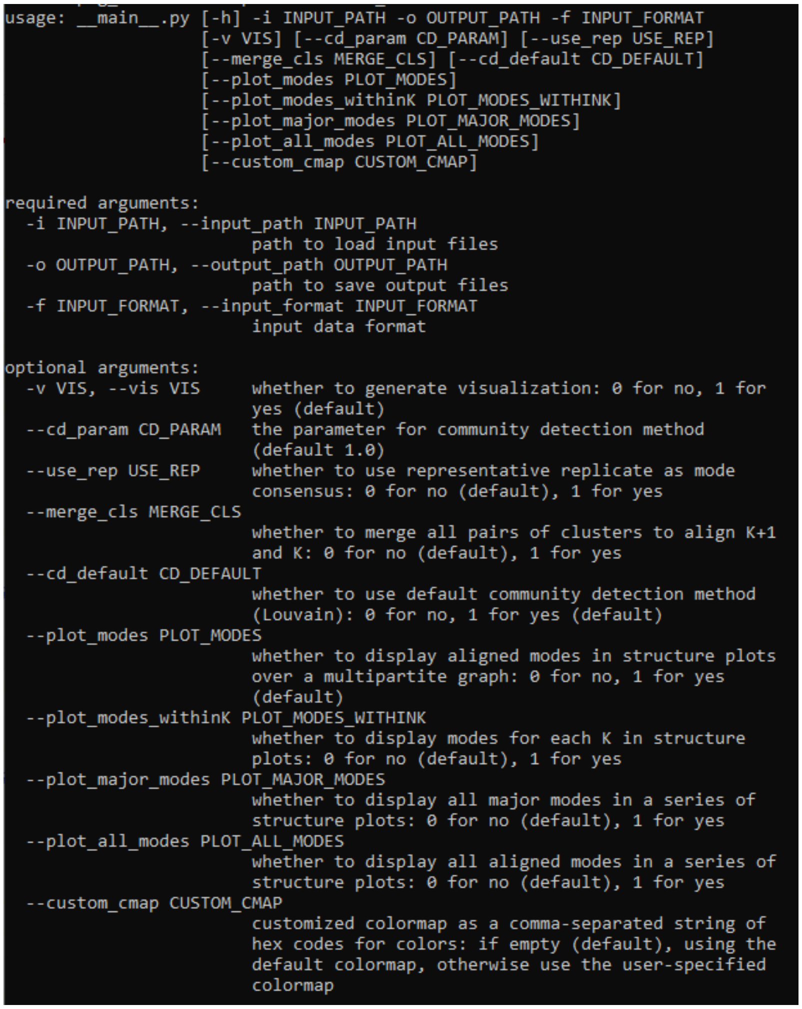

```
usage: __main__.py [-h] -i INPUT_PATH -o OUTPUT_PATH -f INPUT_FORMAT
                   [-v VIS] [--cd_param CD_PARAM] [--use_rep USE_REP]
                   [--merge_cls MERGE_CLS] [--cd_default CD_DEFAULT]
                   [--plot_modes PLOT_MODES]
                   [--plot_modes_withinK PLOT_MODES_WITHINK]
                   [--plot_major_modes PLOT_MAJOR_MODES]
                   [--plot_all_modes PLOT_ALL_MODES]
                   [--custom_cmap CUSTOM_CMAP]

required arguments:
  -i INPUT_PATH, --input_path INPUT_PATH
                        path to load input files
  -o OUTPUT_PATH, --output_path OUTPUT_PATH
                        path to save output files
  -f INPUT_FORMAT, --input_format INPUT_FORMAT
                        input data format

optional arguments:
  -v VIS, --vis VIS     whether to generate visualization: 0 for no, 1 for
                        yes (default)
  --cd_param CD_PARAM   the parameter for community detection method
                        (default 1.0)
  --use_rep USE_REP     whether to use representative replicate as mode
                        consensus: 0 for no (default), 1 for yes
  --merge_cls MERGE_CLS
                        whether to merge all pairs of clusters to align K+1
                        and K: 0 for no (default), 1 for yes
  --cd_default CD_DEFAULT
                        whether to use default community detection method
                        (Louvain): 0 for no, 1 for yes (default)
  --plot_modes PLOT_MODES
                        whether to display aligned modes in structure plots
                        over a multipartite graph: 0 for no, 1 for yes
                        (default)
  --plot_modes_withinK PLOT_MODES_WITHINK
                        whether to display modes for each K in structure
                        plots: 0 for no (default), 1 for yes
  --plot_major_modes PLOT_MAJOR_MODES
                        whether to display all major modes in a series of
                        structure plots: 0 for no (default), 1 for yes
  --plot_all_modes PLOT_ALL_MODES
                        whether to display all aligned modes in a series of
                        structure plots: 0 for no (default), 1 for yes
  --custom_cmap CUSTOM_CMAP
                        customized colormap as a comma-separated string of
                        hex codes for colors: if empty (default), using the
                        default colormap, otherwise use the user-specified
                        colormap
```

Figure 5: Usage prompt of *Clumppling*.

- `plot_modes`, `plot_modes_withinK`, `plot_major_modes`, and `plot_all_modes` (optional): whether to generate corresponding visualizations of the results: 0 for no, 1 for yes. If yes, the corresponding figure(s) will be generated.
  `plot_modes` (default 1): all aligned modes in structure plots over a multipartite graph, where better alignment between the modes is indicated by the darker color of the edges connecting their structure plots, and the cost of optimal alignment is labeled on each edge.
  `plot_modes_withinK` (default 0): A set of figures, one for each number of clusters, with all modes with the same number of clusters in structure plots in one figure.
  `plot_major_modes` (default 0): the major modes of each K aligned in a series of structure plots.
  `plot_all_modes` (default 0) all aligned modes in a series of structure plots.

- `custom_cmap` (optional): a string that is either empty (default "") or contains a list of colors to be used for a customized colormap. If the string is empty then the default colormap will be used. The customized colormap, if provided, should be a list of colors in hex code in a comma-delimited string. An example colormap with five colors looks like `"#FF0000,#FFFF00,#00EAFF #AA00FF,#FF7F00"`. If the provided colormap does not have enough colors for all clusters, colors will be cycled.

The default function can be called by `python -m clumppling` followed by the arguments. You can provide arguments in either of the following ways:

```
python -m clumppling -i INPUT_PATH -o OUTPUT_PATH -f INPUT_FORMAT
```

or

```
python -m clumppling --input_path INPUT_PATH --output_path OUTPUT_PATH --
   input_format INPUT_FORMAT
```

or

```
python -m clumppling --input_path="INPUT_PATH" --output_path="OUTPUT_PATH"
   --input_format="INPUT_FORMAT"
```

where the `INPUT_PATH`, `OUTPUT_PATH`, and `INPUT_FORMAT` denote the user-specific arguments. You may also use a backslash at the end of a line for a line break:

```
python -m clumppling -i INPUT_PATH -o OUTPUT_PATH -f INPUT_FORMAT
```

Details about different input data formats supported by *Clumppling* are described in Section 5.

4.1 Usage of the `merge_cls` parameter

Clumppling offers a "merge" approach for aligning replicates of K and $K+1$, similar to *Pong*'s approach [1], which involves enumerating all possible ways to merge two clusters into one when the number of clusters differs. The number of merging scenarios, which corresponds to the number of alignments to be performed, grows exponentially with K and becomes infeasible to handle. This "merge" approach becomes impossible for replicates with a number of clusters differing by more than one and can be inefficient when the number of combinations to enumerate is large.

Clumppling's default setting uses the "direct" approach which does not have the issues of the "merge" approach and allows the alignment of modes across arbitrary K. The "merge" approach aims to achieve a "cluster-merging optimality," while the "direct" approach does not. The alignment constructed through ILP ensures that each cluster is matched to the most similar cluster, instead of merged together to be close to another cluster, in the other replicate. When *Clumppling* aligns multiple clusters to the same cluster in another replicate, it indicates that these inferred ancestry groups all exhibit high similarity to the ancestry inferred in the replicate with the smaller K.

4.2 Usage of the `cd_param` parameter

There is a trade-off between the granularity of community detection and the number of modes detected. When more modes are identified, each mode tends to have fewer replicates, leading to closer alignment within each mode and higher within-mode similarity. However, in the extreme case, where every replicate becomes a mode and the community detection only finds singletons, the original purpose of community detection is compromised.

When using the default Louvain algorithm, users can choose to adjust the resolution input to the community detection method. Conducting a few preliminary test runs of the program and selecting the parameter value that yields satisfactory results in terms of both within-K performance and the number and size of the modes is recommended. For more challenging datasets, users should consider using a resolution higher than the default value of 1.0. This will prioritize relatively smaller communities and increase their number, thereby generating more fine-scale modes. Since the choice of parameters affects the alignment results, it is advisable to try a few different values when the default setting fails to produce the desired output, e.g., if there are too many singleton modes or if there are noisy modes consisting of replicates with noticeable differences.

5 Input Data Format

The alignment function `clumppling` supports various input formats. It can be used with the alignment of the outputs of widely-used popular population structure inference methods, *Structure*, *Admixture*, *fastStructure*, as well as the general output of any mixed-membership clustering method stored as membership matrices.

5.1 Clustering from *Structure*

To specify the input format as *Structure* results files, you need to use the following argument for the function `clumppling`

```
--input_format structure  # or -f structure
```

The `input_path` should point to the "Results" folder that stores the output files of *Structure*. This folder should be automatically generated when you run a *Structure* job. For instance, when you run *Structure* on a project with a parameter set named `xxx` for 100 runs, this folder will contain files with names `xxx_run_1_f` to `xxx_run_100_f`. Specifically, *Clumppling* will extract the membership coefficients from the section "Inferred ancestry of individuals:" in these files.

5.2 Clustering from *Admixture*

To specify the input format as *Admixture* result files, you need to use the following argument for the function `clumppling`

```
--input_format admixture  # or -f admixture
```

The `input_path` should point to a folder that stores the output files of *Admixture*, namely, those ending with "K.Q", where K is the number of clusters. These *Admixture* output files record the inferred ancestry fractions in membership matrices. It is fine to leave other files (e.g., the ".P" files), if any, in the same folder. The program will ignore all files not with the corresponding extension. E.g., if there are 50 ".Q" files, then *Clumppling* will attempt to align these 50 runs of the clustering.

It can also accept the manipulated *Admixture* result files ending with ".indivq" in which additional individual information is stored in the same manner as in *Structure* files, i.e., the additional individual information is available in the leading 4 columns (space-delimited, before the column with ":") before the membership coefficients, representing the individual index, the individual label, the percentage of missingness, and the population label.

5.3 Clustering from ***fastStructure***

To specify the input format as *fastStructure* results files, you need to use the following argument for the function `clumppling`

```
--input_format fastStructure
```

The `input_path` should point to the folder that stores the output files of *fastStructure*. Specifically, this folder needs to contain the output ".K.meanQ" files, where K is the number of clusters for the run. The ".meanQ" contains the posterior mean of admixture proportions. If the folder contains other files, like ".meanP", ".varP", and ".varQ", the program will simply ignore them, so there is no need to filter those files out after running *fastStructure*. You simply need to use the output directory of *fastStructure* as the `input_path` for *Clumppling*.

5.4 General Membership Matrices from Any Clustering

Clumppling also accepts input as general membership matrices, regardless of the clustering methods used for generating them. To specify such input format, which we term "general Q matrices", you need to use the following argument for the function `clumppling`

```
--input_format generalQ
```

The file format in the folder that `input_path` is pointing to should be the same as that for the input data coming from *Admixture* output, the Q files. The files should end with ".Q". There is no need to include K in the file name, as the program will automatically detect the number of clusters when loading the clustering results. Each ".Q" file should contain the space-delimited membership matrix of a run. For instance, if there are 100 individuals clustered into 4 ancestries, then each row should contain 4 values, separated by space and summing up to 1, which correspond to the membership coefficients of an individual, and there should be 100 rows in the file.

6 Output Files

The main function `clumppling` generates outputs in a folder specified by `output_path` and zips them into a zip file "OUTPUT_PATH.zip". The folder will include six subfolders and an output file "output.log". An "output.log" file contains all the output to the console when running *Clumppling*, which also documents the run time. The contents of the six subfolders are described in detail in the following subsections.

Alignment pattern. In the output files, the optimal alignment between a pair of replicates (or modes) is recorded as a space-separated group of numbers. For instance, if Replicate 1 and Replicate 2, each with five clusters, have the alignment pattern `4 2 1 5 3`, the five clusters in Replicate 2 should be matched to clusters 4, 2, 1, 5, and 3 in Replicate 1, respectively. The number of clusters in Replicate 2 will always be no less than that in Replicate 1. Multiple clusters in Replicate 2 may be matched to the same cluster in Replicate 1,

6.1 The "input" folder

The "input" folder contains the following files:

- ".Q" files of membership matrices, which are the input clustering results loaded and processed by *Clumppling*. The naming of the".Q" files, which are the labels of the replicates used by *Clumppling*, follows the pattern of `"1_K5R2.Q"`, where 1 is the index of the clustering results used in by *Clumppling*, 5 is the number of clusters, and 2 is the index of this replicate in all replicates with this number of clusters.
- A comma-delimited "input_files.txt" file with two fields. The first field is the name of processed ".Q" files and the second field is the corresponding name of the original input file.
- A "ind_info.txt" file if the input data format provides population labeling of the individuals. It contains auxiliary information on the individuals like identification label and population, where the order of the individuals is the same as that in the membership matrices.

6.2 The "alignment_withinK" folder

The "alignment_withinK" folder stores the results of all pairwise alignment of replicates with the same number of clusters. It contains one comma-delimited text file for each K value in the input. Each file contains three fields: three fields: `Replicate1-Replicate2`, `Cost`, and `Alignment`. `Replicate1-Replicate2` records the labels of the two replicates. `Cost` records the cost of optimal alignment between the two replicates. `Alignment` records the optimal alignment pattern between the two replicates in a space-separated group of numbers. Each line in the file, except for the header, corresponds to the alignment results of a pair of replicates with the specified number of clusters.

For example, the following line

```
151_K5R1-152_K5R2,0.01769820597265037,4 2 1 5 3
```

records the alignment results between replicate `151_K5R1` and replicate `152_K5R2`. The optimal alignment pattern between them is `4 2 1 5 3`, and under this optimal alignment, the cost between the aligned replicates is `0.01769820597265037`.

6.3 The "modes" folder

The "modes" folder contains the following files:

- ".Q" files of the consensus membership matrices of all modes detected. The naming of the".Q" files, which are the labels of the modes used by *Clumppling*, follows the pattern of `"K5M1_avg.Q"`, where 5 is the number of clusters, 1 is the index of this mode in all modes with this number of cluster, and `mean` stands for using the mean memberships as the consensus of a mode. An alternative is `rep`, which tells the program to use the representative replicate as the consensus of a mode.
- A comma-delimited "mode_alignments.txt" file with four fields: `Mode`, `Representative`, `Replicate`, and `Alignment`. A line in the file looks like this:

  ```
  K5M1,152_K5R2,151_K5R1,3 2 5 1 4
  ```

  `Mode` records the label of the mode, e.g., `K5M1`. `Representative` records the label of the representative replicate of the mode, e.g., `152_K5R2`. `Replicate` records the label of the focal replicate , e.g., `152_K5R1`. `Alignment` records the space-separated alignment pattern between the representative and the focal replicates.

- A comma-delimited "mode_stats.txt" file with five fields: `Mode`, `Representative`, `Size`, `Cost`, and `Performance`. `Mode` and `Representative` are the same as in "mode_alignments.txt". `Size`, `Cost`, and `Performance` record the size (number of clusters) of the mode, the averaged cost of the alignment between all pairs of clusters in the mode (optimal objective of Eq. 12 in the paper), and the averaged performance measure, calculated as the H' similarity (Eq. 18 in the paper), of all the pairwise alignments in the mode. A lower cost and a higher performance value indicate a better alignment quality for the mode detected.

- A comma-delimited "mode_average_stats.txt" file with five fields: `K`, `Size`, `NS-Size`, `Cost`, and `Performance`. `K` is the number of clusters. `Size` is the total number of replicates. `NS-Size` is the number of replicates not belonging to singleton modes (i.e., modes with only one replicate). `Cost` is the weighted average of the cost of all modes not including the singletons, weighted by the mode size (using a calculation similar to Eq. 20 on the optimal objective of Eq. 12 in the paper). `Performance` is the weighted average of the performance measure (H' similarity) of all modes not including the singletons, weighted by the mode size (Eq. 20 in the paper).

6.4 The "alignment_across_K" folder

The "alignment_across_K" folder contains two files for each type of consensus membership of the mode, specified by the suffixes `"_avg"` and `"_rep"`.

- A comma-delimited "alignment_acrossK.txt" file with three fields: `Mode1-Mode2`, `Cost`, and `Alignment`. Each line in the file, except for the header, corresponds to the alignment results of a pair of modes. `Mode1-Mode2` records the labels of the two modes, where the second mode has a number of clusters no less than the first mode. `Cost` records the cost of optimal alignment between the two modes. `Alignment` records the optimal alignment pattern between the two modes in a space-separated group of numbers.

- A comma-delimited "best_pairs_acrossK.txt" file with five fields: `Best Pair`, `Cost`, `Alignment`, `Separate-Cluster Cost`, and `Separate-Cluster Alignment`. `Best Pair` is the best pair of modes between adjacent K in terms of the alignment performance, e.g., `K4M1-K5M1`. `Cost` and `Performance` record the cost and performance measure of optimal alignment between this pair of modes by merging the clusters in Mode 2 that get matched to the same cluster in Mode 1 and comparing two membership matrices of the same size after the merging. `Separate-Cluster Cost` and `Separate-Cluster Performance` record the cost and performance measurement that is calculated by comparing each pair of clusters that are matched and summing over the value for all clusters. For two or more clusters from Mode 2 that are matched to the same cluster in Mode 1, the average cost and performance of them are considered.

6.5 The "modes_aligned" folder

The "modes_aligned" folder contains the following files:

- ".Q" files of the consensus membership matrices of all modes that are aligned across different numbers of clusters. The best pair of modes for each pair of adjacent K values are used as anchors. The rest of the modes with the same K are aligned to the anchor according to results in "mode_alignments.txt" (in the "modes" folder), then the anchors are aligned according to results in the "alignment_across_K" folder. The naming of the".Q" files follows the pattern of `"K5M1_aligned_avg.Q"`, where the suffix `"_avg"` denotes the type of consensus membership used.

- A comma-delimited "all_modes_alignment.txt" file for each type of consensus membership of the mode specified by the suffixes `"_avg"` and `"_rep"`. A line in the file looks like this

  ```
  K5M1:4 3 2 1 5
  ```

  where before the colon is the mode, and after the colon is the permutation pattern that is used to generate the aligned mode in "modes_aligned" from the unaligned ones in the "modes" folder.

6.6 The "visualization" folder

The "visualization" folder contains all the output figures if the corresponding parameters are set to `T`.

- "colorbar.png" is the colorbar showing the colormap used in visualization, where each color represents a distinct cluster, indexed from 1 to K. The colormap may be specified by the user by setting the `custom_cmap` parameters.

- "modes_aligned_multipartite_avg.png" and "modes_aligned_multipartite_rep.png" are the main visual output of the program. They plot the aligned modes over a multi-partite graph picturing the alignment relationships across different K. The modes are plotted in stacked bar plots, where the number of replicates in each mode is marked on the top right corner of each bar plot. Better alignment between the modes is indicated by the darker color of the edges connecting their bar plots, and the cost of optimal alignment is labeled on each edge. These figures will only be generated if the parameter `plot_modes` is `1`.

- "major_modes_aligned_avg.png" and "major_modes_aligned_rep.png" plot the memberships in a series of stacked bar plots for the major modes for all K, ordering from the smallest K to the largest. These figures will only be generated if the parameter `plot_major_modes` is `1`. An example is shown in Figure 6.

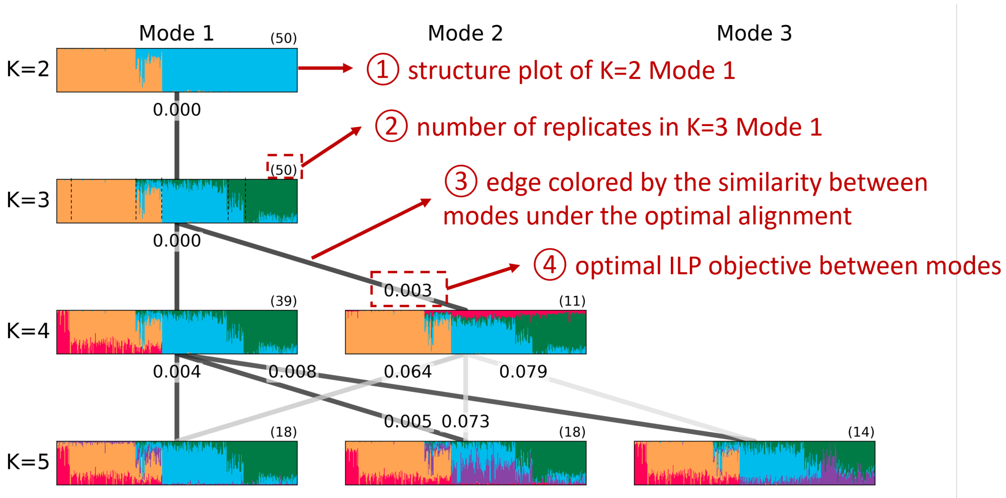

Figure 6: Visualization of main alignment result example (Figure 1 in the main text): modes are depicted in structure plots and connected by edges indicating pairwise alignment. Each row corresponds to a K value and each column corresponds to a mode.

- "modes_cost_hist.png" plots the histogram of edge weights in each community (mode) identified during the mode detection. The edge weights are pairwise dissimilarities under optimal alignment between all replicates within each mode, and their distribution indicates the variability within each mode. The mean edge weight is marked by the red dashed line on each histogram. Modes are laid out in the same way as in the main visual output. An example is shown in Figure 7.

- One figure for each K for each type of consensus membership, with the name following "K5_modes_avg.png". The figure plots the memberships in a series of stacked bar plots for all aligned modes of the specific K. These figures will only be generated if the parameter `plot_modes_withinK` is `1`.

- "modes_aligned_avg.png" and "modes_aligned_rep.png" plot the memberships in a series of stacked bar plots for all aligned modes for all K, ordering from the smallest K to the largest and mode indexes from small to large. These figures will only be generated if the parameter `plot_modes` is `1`.

In all the figures, each color represents a cluster, where the colors are specified by the customized colormap if `custom_cmap` is provided.

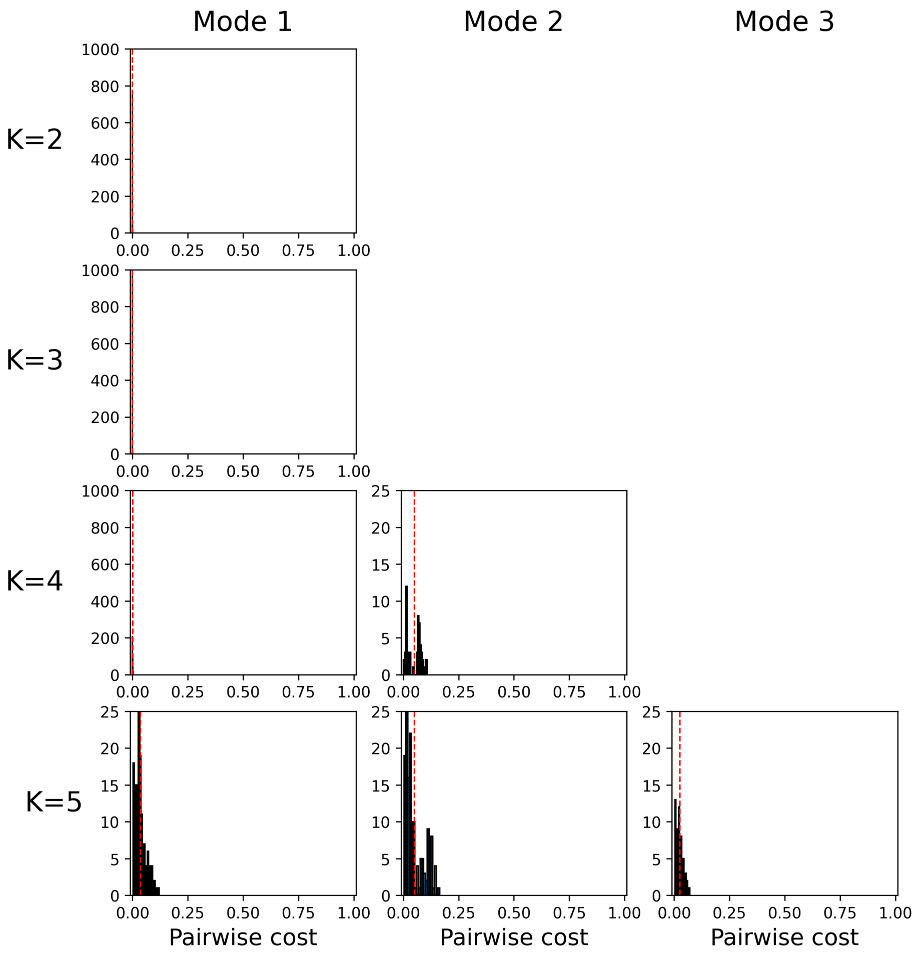

Figure 7: Visualization of mode variability example: histogram of pairwise dissimilarities between all replicates within a mode is shown for each mode, where modes are laid out in the same way as in Figure 6. The mean pairwise dissimilarity is marked by the red dashed line.

7 Examples

To run the demonstrations, make sure that your current directory is the "Clumppling" directory and that the folder with input files is put under "Clumppling/input". If you previously downloaded the data files as a zip file, unzip them to a folder with the same name. You may check your current working directory by running the command `pwd` (not working on some Windows terminal, use `cd` instead).

The commands provided in this section can be used to run the examples. Alternatively, they can be run through the bash scripts which contain all the commands. To run the bash scripts, you need to install Bash on the system if it is not already there. The example bash scripts to run the demonstrations, as well as the default parameter files, are under the directory "Clumppling/scripts".

7.1 Datasets

To demonstrate the usage of this package, we use several published genetic datasets, and run clustering algorithms on those datasets to get the input to our alignment framework.

7.1.1 Human genotype data

The human genotype data contains genotype information of 399 individuals, including 44 individuals in the admixed population of Cape Verde and selected West African and Western European populations [8]. *Admixture* was run

50 times independently for each K value from 2 to 5, resulting in a total of 200 clustering replicates. This dataset is used to demonstrate the alignment of *Admixture* output files.

7.1.2 Chicken microsatellite data

The chicken microsatellite data comprises 27 microsatellite loci genotyped in 600 individuals from 20 chicken populations [6]. We run *Structure* [4] on the full set of loci for 20 runs for each K from 17 to 21, then align all the 100 replicates using *Clumppling*. This dataset is used to demonstrate the alignment of *Structure* output files.

7.2 Putting the data files under the correct directory

If you download the data files separately, unzip them and put them under a folder called "input" under your current working directory.

If you do not know your current path, you can run `pwd` to see it in the command window (not working on some Windows terminals, use `cd` instead).

For example, if your current working path is `C:/Users/USERNAME/Clumppling`, then the path for the example files should be `C:/Users/USERNAME/Clumppling/input`, and the path for the Cape Verde example data should be `C:/Users/USERNAME/Clumppling/input/capeverde`.

The Cape Verde data files will be located in `input/capeverde` under your current path. I.e., When you run `ls input/capeverde` (or `dir input/capeverde` for Windows), you should see a list of Cape Verde data files ("CAPEVERDE_Rep1.2.indivq", etc.)

If you install the package after downloading the entire directory, you should have all the data files ready if your current directory is the downloaded directory, e.g., `C:/Users/USERNAME/Clumppling`.

7.3 Running `clumppling` on Cape Verde data

To run align the clustering outputs on the Cape Verde dataset which come from different runs of the program *Admixture*, you can run the following command (for default community detection and visualization settings)

```
python -m clumppling --input_path input/capeverde --output_path output/
    capeverde_default --input_format admixture
```

Alternatively, you may write the command in a bash script and run the bash script. For example, when you are in the "Clumppling" directory, you can run the same example using the bash script in "run_ex1_default' as follows

```
bash scripts/run_ex1_default.sh
```

This example will generate the following outputs in the console.

The results, including figures and text files, will be saved in the output folder. For instance, the file "modes_stats.txt" under the "modes" folder look like

```
Mode,Representative,Size,Cost,Performance
K2M1,3_K2R3,50,8.043782926676283e-14,0.9999997631447923
K3M1,59_K3R9,50,3.0678737343358222e-09,0.9999591847910263
K4M1,145_K4R45,39,0.0007341008438339235,0.9788891519804838
K4M2,112_K4R12,11,0.04944348952966262,0.7918294897008602
K5M1,179_K5R29,18,0.03704964921858028,0.8188413674149748
K5M2,165_K5R15,18,0.050741851309627946,0.7939365967826061
K5M3,189_K5R39,14,0.02550705761774155,0.847881111372104
```

7.4 Running `clumppling` on chicken data

To run align the clustering outputs on the chicken dataset which comes from different runs of the program *Structure*, you can run the following command (for default visualization settings and a customized community detection parameter)

```
python -m clumppling --input_path input/chicken --output_path output/
    chicken_default --input_format structure --cd_param=1.05
```

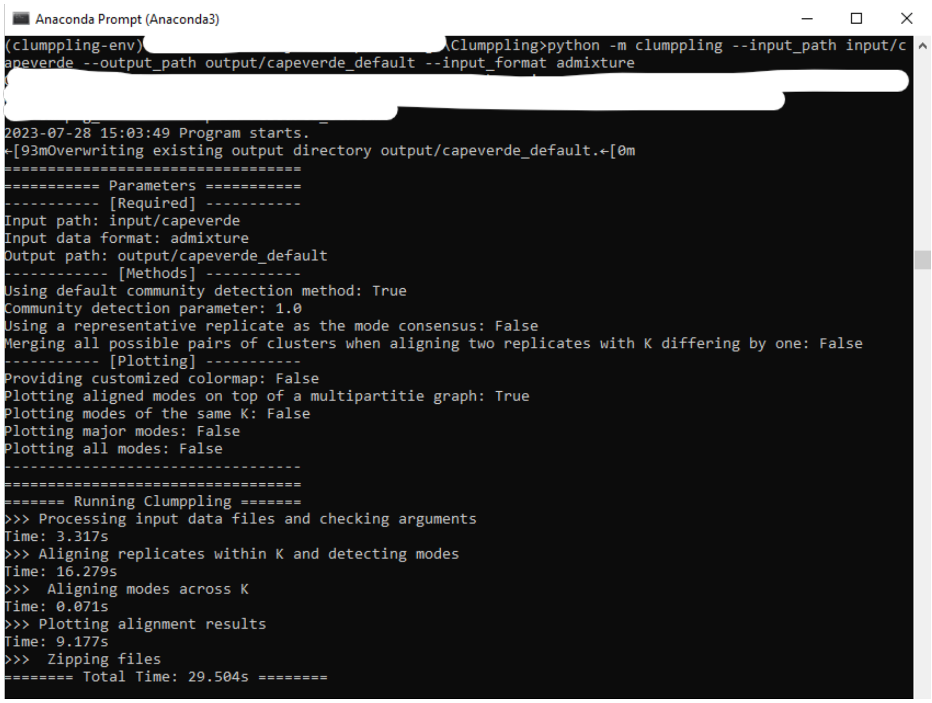

Figure 8: Output log of the Cape Verde example.

Alternatively, you may write the command in a bash script and run the bash script. For example, when you are in the "Clumppling" directory, you can run the same example using the bash script in "run_ex2_default.sh" as follows

```
bash scripts/run_ex2_default.sh
```

This example will generate the following outputs in the console:

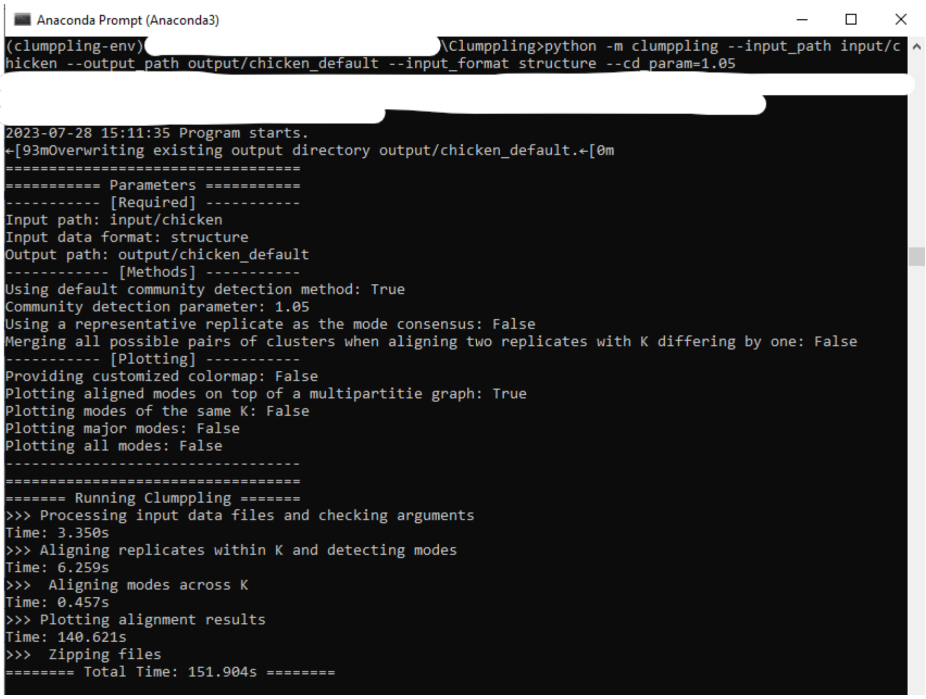

Figure 9: Output log of the chicken example.

The results, including figures and text files, will be saved in the output folder. For instance, the first five lines of the file "modes_stats.txt" under the "modes" folder look like

```
Mode,Representative,Size,Cost,Performance
K17M1,1_K17R1,6,0.048931782944444425,0.7918254748602578
K17M2,14_K17R14,5,0.06331773349999999,0.7626459575134236
K17M3,9_K17R9,5,0.12416171116666663,0.6498140751787831
K17M4,2_K17R2,4,0.09807973263888886,0.6932660053254581
```

7.5 Running `clumppling` on outputs with a non-consecutive number of clusters

To demonstrate *Clumppling*'s ability to align replicates when the number of clusters are not consecutive, we provide an additional example with a subset of runs from the chicken data, with $K = 17$, 19, and 21. The command stays the same except for the input path which should now be `input/chicken_gapK`.

8 Additional Functionalities

Besides aligning different replicates generated by a clustering model and detecting modes, *Clumppling* provides two extensions to compare memberships of the same individuals generated using different models, and to align memberships of different sets of individuals when they have population labels.

8.1 `clumppling.diffModel`: aligning memberships of the same individuals from different models

When the memberships are generated using different models, and within each model, there are several replicates with different numbers of clusters K, we may not want to align all replicates and aggregate all into consensus memberships for each K. Instead, we want to obtain the consensus for results generated using each model, then directly compare all the models. *Clumppling* adds this functionality by first detecting modes for each model, then aligning modes with the same K from different models to the first mode from the first model, where the order of the models can be manipulated by users. In this case, *Clumppling* provides the function `clumppling.diffModel` to first perform within-model alignment of the clustering results, then perform across-model alignment, and output intuitive visualizations for a direct comparison of clustering results generated based on different models.

8.1.1 How to run `clumppling.diffModel`

`clumppling.diffModel` can be used to call the program to align memberships of the same individuals from different models. By running the command

```
python -m clumppling.diffModel -h
```

you will get

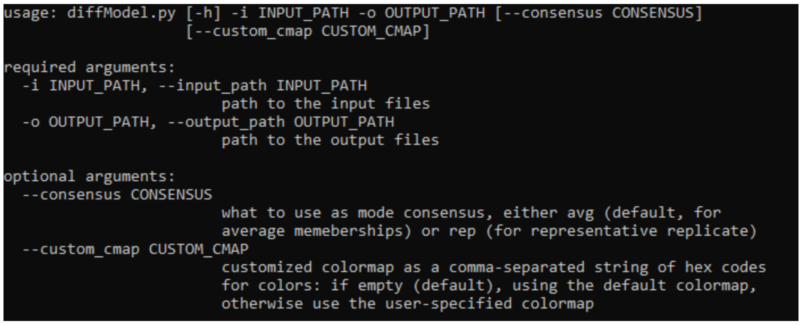

```
usage: diffModel.py [-h] -i INPUT_PATH -o OUTPUT_PATH [--consensus CONSENSUS]
                    [--custom_cmap CUSTOM_CMAP]

required arguments:
  -i INPUT_PATH, --input_path INPUT_PATH
                        path to the input files
  -o OUTPUT_PATH, --output_path OUTPUT_PATH
                        path to the output files

optional arguments:
  --consensus CONSENSUS
                        what to use as mode consensus, either avg (default, for
                        average memeberships) or rep (for representative replicate)
  --custom_cmap CUSTOM_CMAP
                        customized colormap as a comma-separated string of hex codes
                        for colors: if empty (default), using the default colormap,
                        otherwise use the user-specified colormap
```

Figure 10: Usage prompt of *Clumppling.diffModel*.

This function takes in two required arguments and two optional arguments:

- `input_path` (`-i`, required): path to the parent folder that contains the alignment results of each model
- `output_path` (`-o`, required): path to the folder that the user wants to save the results of alignment
- `consensus` (optional, default: `"avg"`): Either `"avg"` or `"rep"`, indicating which consensus membership of the modes is to be used for the alignment. `"avg"`: average memberships of replicates in the mode. `"rep"`: a representative replicate from the mode.
- `custom_cmap` (optional, default: the empty string“”): The customized colormap, which should be a list of colors in hex code in a comma-delimited string. If the string is empty, then the default colormap will be used. An example colormap with five colors looks like `"#FF0000 #FFFF00 #00EAFF #AA00FF #FF7F00"`. If the provided colormap does not have enough colors for all clusters, colors will be cycled.

The default function can be called by

```
python -m clumppling.diffInd --input_path
   PARENT_DIRECTORY_OF_ALIGNMENT_RESULTS --output_path OUTPUT_PATH
```

8.1.2 Outputs

The function `clumppling.diffModel` generates outputs in a folder specified by `output_path` containing three files and zips them into a zip file “OUTPUT_PATH.zip”. Besides the inputs to `clumppling.diffModel` that come from the results of `clumppling`, the folder contains four files:

1. An “output.log” file with all the output to the command window when running `clumppling.diffModel`. It documents the run time.
2. A comma-delimited “multiple_inputs.txt” file with two fields. The first field is the index and the second field is the corresponding name of the input (model), where the inputs are the alignment results from `clumppling`. The name of the input needs to be the same as the name for the output directory of `clumppling`.
3. A comma-delimited “multiple_alignment.txt” file with five fields: `K`, `input1_index`, `input2_index`, `optimal_obj`, and `alignment`. `K` is the number of clusters. `input1_index` and `input2_index` are the indices of two inputs (models). `optimal_obj` and `alignment` is the objective of the ILP and the pattern of the optimal alignment that is identified via ILP between the first modes (`"M1"`) of the specified K for the two inputs. For instance, a line

   ```
   5,1,2,0.44606332580777075,4 3 1 5 2
   ```

   means that `K5M1` of the first input and `K5M1` of the second input are optimally aligned by the pattern `4 3 1 5 2` with an objective of `0.44606332580777075`.
4. A figure “aligned_all.png” showing the modes of different models side by side. The models are aligned by the first mode of each model, and ordered from left to right in the same way that their results are in the parent directory specified by `input_path`. The modes are ordered from smallest K to largest K from top to bottom.

8.1.3 Example

Dataset An HGDP (Human Genome Diversity Cell Line Panel) human microsatellite dataset is used to demonstrate the alignment of replicates obtained from different clustering models

This first HGDP dataset, analyzed in [2], contains a sample of 978 individuals each genotyped at 791 microsatellite (STR) loci, including 13 CODIS loci. This is used to demonstrate the use of the `diffModel` method.

Recall that by “different models”, we follow the definition in [3] to denote the scenarios where clustering results are obtained from different programs, different model choices, or different subsets of genetic markers across the same data set. We run *Structure* on the first HGDP dataset using two different models: one based on all 791 loci, and one based on 13 CODIS loci. We run *Structure* 10 times for each K from 2 to 6 using each model, using a burn-in period of 10,000 steps followed by 10,000 iterations. The two models generate clustering results that have intrinsically different “alignment cost”, as discussed in [9], with the one based on fewer loci having replicates with much higher variation and being much harder to align. Aligning replicates from the two different models, therefore, can also be challenging. We demonstrate here that *Clumppling* can help with this use case.

Commands The following example aligns memberships of the same individuals from running *Structure* on the full set of 791 loci and a selected set of 13 CODIS loci in the first HGDP dataset.

```
python -m clumppling -i input/diffModel/791loci -o output/diffModel/791loci
    -f structure --cd_default 0
python -m clumppling -i input/diffModel/13loci -o output/diffModel/13loci -f
    structure --cd_default 0
python -m clumppling.diffModel -i output/diffmodel -o output/diffmodel
```

Note that the output paths for the two calls of the main function, need to be subfolders of the same directory "output/diffModel", which is the input path for `clumppling.diffModel`. This example considers the clustering results using only two different models, but *Clumppling* can support the alignment for more than two models, as long as their within-model alignment results are put under the same directory for the `input_path` of `diffModel`. Alternatively, you can run the example by

```
bash scripts/ex4.sh
```

Results The alignment results are shown in Figure 11, which is saved in a figure titled "aligned_all.png" in the output directory. *Clumppling* first aligns the replicates and detects the modes for each model, then aligns modes between models and shows them side by side.

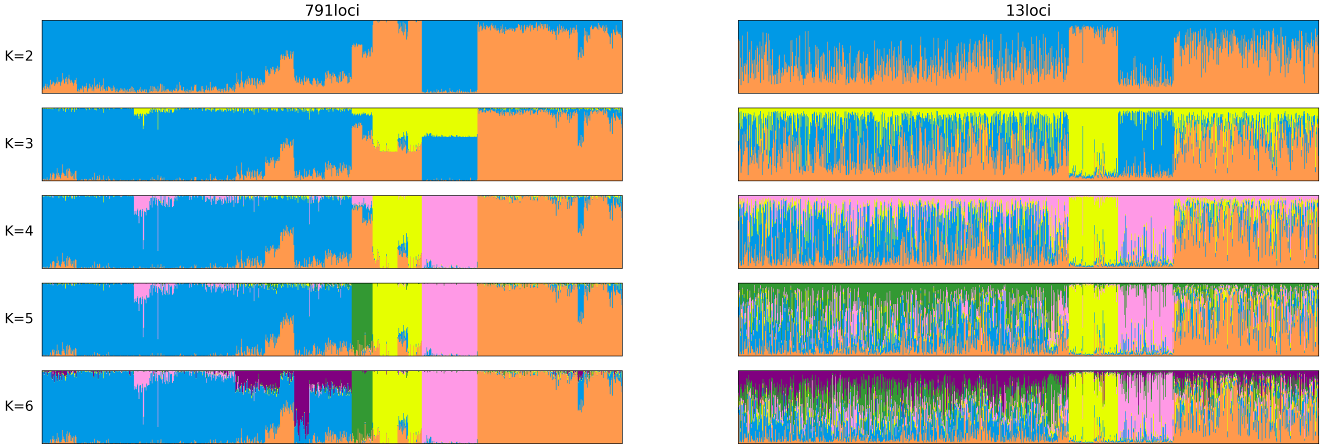

Figure 11: Aligned modes for population structure inferred from models using different loci in HGDP data: (left) the full set of 791 loci and (right) the 13 CODIS loci.

8.2 `clumppling.diffInd`: aligning memberships of different individuals from (possibly different) models

When the memberships of populations are generated using different sets of individuals, for example, subsets of different sample sizes from the same populations, the membership matrices Q will no longer be of the same size. In this case, *Clumppling* provides the function `clumppling.diffInd` to perform the alignment based on their population-wise average memberships and compare the alignment results side by side for each set of individuals. Note that the alignment of clustering results from a different set of individuals is only appropriate when the population label, or some other types of labels that encode some grouping information so that individuals with the same label show relative consistency in their membership coefficients inferred, can be assigned to each individual. This is because the alignment across different individual sets relies on calculating population-wise membership coefficients from individual membership coefficients and then aligning the replicates based on the population-wise memberships.

Taking the same notations from the manuscript, we denote the number of modes detected for each K as m_K, and denote the modes as M_K^1 through $M_K^{m_K}$, with $M_K^i \cap M_K^j = \emptyset$ for all $i, j \in [m_k]$. The corresponding consensus memberships are $\bar{Q}_K^1$ through $\bar{Q}_K^{m_K}$ with size $N \times K$. Suppose a first set of outputs A have modes $\{({}^A M_K^\ell, {}^A \bar{Q}_K^\ell) : \ell \in [m_K]\}_{K \in \mathcal{K}}$ where N_A individuals are from populations $[PA]$, and a second set of outputs B

have modes $\left\{({}^{B}M_K^{\ell}, {}^{B}\bar{Q}_K^{\ell}) : \ell \in [m_K]\right\}_{K\in\mathcal{K}}$ where N_B individuals are from populations $[PB]$. Then for each K where these outputs overlap, we select the consensus memberships ${}^{A}\bar{Q}_K^1$ and ${}^{B}\bar{Q}_K^1$, and compute the population-wise average memberships as

$$ {}^{A}\mathcal{Q}_{jk} = \sum_{i\in[j]} {}^{A}\bar{Q}_{ik}^1 \tag{1} $$

and ${}^{B}\mathcal{Q}_{jk}$, similarly, for each population $j \in [PA]\bigcup[PB]$ and each $k \in [K]$. Both population-wise membership matrices ${}^{A}\mathcal{Q}$ and ${}^{B}\mathcal{Q}$ have size $|[PA]\bigcup[PB]| \times K$.

Pairwise alignment between ${}^{A}\bar{Q}_K^1$ and ${}^{B}\bar{Q}_K^1$ is then done by ILP using their population-wise average memberships ${}^{A}\mathcal{Q}$ and ${}^{B}\mathcal{Q}$ and a modified between-cluster distance weighted by population sizes. That is, the dissimilarity between two clusters is modified to be

$$ C'(q_i, p_j) = \sum_{\ell=1}^{|[PA]\bigcup[PB]|} \frac{N_{A,\ell} + N_{B,\ell}}{N'_A + N'_B} |Q_{\ell i} - P_{\ell j}|, \tag{2} $$

where N'_A and N'_B correspond to the total number of individuals in the overlapped populations between A and B, and $N_{A,\ell}$ and $N_{B,\ell}$ correspond to the number of individuals in population ℓ. The rest of the alignment follows the same procedure as in the individual membership alignment. The modes $\{{}^{A}\bar{Q}_K^{\ell}\}_{\ell\in[m_K]}$ and $\{{}^{B}\bar{Q}_K^{\ell}\}_{\ell\in[m_K]}$ for each K are then aligned based on the optimal alignment between ${}^{A}\bar{Q}_K^1$ and ${}^{B}\bar{Q}_K^1$. *Clumppling* presents the aligned modes based on each set of outputs side by side, with modes of the same K starting at the same row.

8.2.1 How to run `clumppling.diffInd`

`clumppling.diffInd` can be used to call the program to align memberships of different individuals. By running the command

```
python -m clumppling.diffInd -h
```

you will get

```
usage: diffInd.py [-h] -i INPUT_PATH -o OUTPUT_PATH [--consensus CONSENSUS]
                  [--custom_cmap CUSTOM_CMAP]

required arguments:
  -i INPUT_PATH, --input_path INPUT_PATH
                        path to the input files
  -o OUTPUT_PATH, --output_path OUTPUT_PATH
                        path to the output files

optional arguments:
  --consensus CONSENSUS
                        what to use as mode consensus, either avg (default, for
                        average memeberships) or rep (for representative replicate)
  --custom_cmap CUSTOM_CMAP
                        customized colormap as a comma-separated string of hex codes
                        for colors: if empty (default), using the default colormap,
                        otherwise use the user-specified colormap
```

Figure 12: Usage prompt of *Clumppling.diffInd*.

This function takes in two required arguments and two optional arguments:

- `input_path` (`-i`, required): path to the parent folder that contains the alignment results of each set of input
- `output_path` (`-o`, required): path to the folder that the user wants to save the results of alignment
- `consensus` (optional, default: `"avg"`): Either `"avg"` or `"rep"`, indicating which consensus membership of the modes is to be used for the alignment. `"avg"`: average memberships of replicates in the mode. `"rep"`: a representative replicate from the mode.
- `custom_cmap` (optional, default: the empty string“”): The customized colormap, which should be a list of colors in hex code in a comma-delimited string. If the string is empty then the default colormap will be used. An example colormap with five colors looks like `"#FF0000 #FFFF00 #00EAFF #AA00FF #FF7F00"`. If the provided colormap does not have enough colors for all clusters, colors will be cycled.

The default function can be called by

```
python -m clumppling.diffModel --input_path
   PARENT_DIRECTORY_OF_ALIGNMENT_RESULTS --output_path OUTPUT_PATH
```

Note that running `clumppling.diffInd` requires some labeling information of the individuals, for instance, their populations. If the population label is provided as input to *Structure*, the labeling can be automatically extracted when running `clumppling` with `input_type="structure`. If the population label is provided in the ".indivq" files as the outputs of *Admixture*, then it will also be extracted automatically. However, currently *Clumppling* does not support automatic extraction of individual information with other input data format, so a comma-delimited file named "ind_info.txt" needs to be manually added to the "data" directory in the outputs of `clumppling` with a field "Pop" storing the population label information (more in Section 6). This space-delimited file should have the column names of the individual information data on the first line. There must be a column named "Pop" which contains the population label of the individuals. The individuals must be of the same order as they appear in the clustering membership matrices.

8.2.2 Outputs

The function `clumppling.diffInd` generates outputs in a folder specified by `output_path` containing three files and zips them into a zip file "OUTPUT_PATH.zip". Besides the inputs to `clumppling.diffInd` that come from the results of `clumppling`, the folder contains four files:

1. An "output.log" file with all the output to the command window when running `clumppling.diffInd`. It documents the run time.

2. A comma-delimited "popwise_inputs.txt" file with two fields. The first field is the index and the second field is the corresponding name of the input (set of individuals), where the inputs are the alignment results from `clumppling`. The name of the input needs to be the same as the name for the output directory of `clumppling`.

3. A comma-delimited "popwise_alignment.txt" file with five fields: `K`, `input1_index`, `input2_index`, `optimal_obj`, and `alignment`. `K` is the number of clusters. `input1_index` and `input2_index` are the indices of two inputs (sets of individuals). `optimal_obj` and `alignment` is the objective of the ILP and the pattern of the optimal alignment that is identified via ILP between the first modes (`"M1"`) of the specified K for the two inputs. For instance, a line

   ```
   5,2,3,0.013166727300769145,2 1 5 3 4
   ```

   means that `K5M1` of the second input and `K5M1` of the third input are optimally aligned by the pattern `2 1 5 3 4` with an objective of `0.013166727300769145`.

4. A figure "aligned_all.png" showing the modes of clustering results using different sets of individuals side by side. The different sets are aligned by the first mode of each, and ordered from left to right in the same way that their results are in the parent directory specified by `input_path`. The modes are ordered from smallest K to largest K from top to bottom.

8.2.3 Example

Dataset A second HGDP dataset is used to demonstrate the alignment of replicates generated on different sets of labeled individuals. The second HGDP dataset, analyzed in [7], contains 377 autosomal microsatellites in 1,056 individuals from 52 populations. This is used to demonstrate the use of the `diffInd` method.

Using the second HGDP dataset, we follow [5] and subset 50, 100, and 200 individuals from each of the seven regions, resulting in 339, 639, and 1,005 individuals in total, respectively. We then run *Structure* 5 times for each K from 2 to 6 on each of the three sets of individuals. Again, we use a burn-in period of 10,000 steps and 10,000 iterations.

Commands The following example aligns memberships output by *Structure* on the second HGDP dataset, where a subset of 50, 100, or 200 individuals for each population is sampled to be input into *Structure*.

```
python -m clumppling -i input/diffInd/ind200 -o output/diffInd/ind200 -f
   structure --cd_default 0
```

```
python -m clumppling -i input/diffInd/ind100 -o output/diffInd/ind100 -f
    structure --cd_default 0
python -m clumppling -i input/diffInd/ind50 -o output/diffInd/ind50 -f
    structure --cd_default 0
python -m clumppling.diffInd -i output/diffInd -o output/diffInd
```

Similar to `clumppling.diffModel`, the output paths for the three calls of the main function, all need to be subfolders of the directory "output/diffModel", which is the input path for `clumppling.diffInd`. This example considers the clustering results using three sets of different individuals, but *Clumppling* can support the alignment for more, as long as their own alignment results are put under the same directory for the `input_path` of `diffInd`.

Alternatively, you can run the example by

```
bash scripts/ex5.sh
```

Results The alignment results are shown in Figure 13, which is saved in a figure titled "aligned_all.png" in the output directory. Based on the population-wise memberships, the population structure generated using different sample sizes of data can be aligned and compared intuitively.

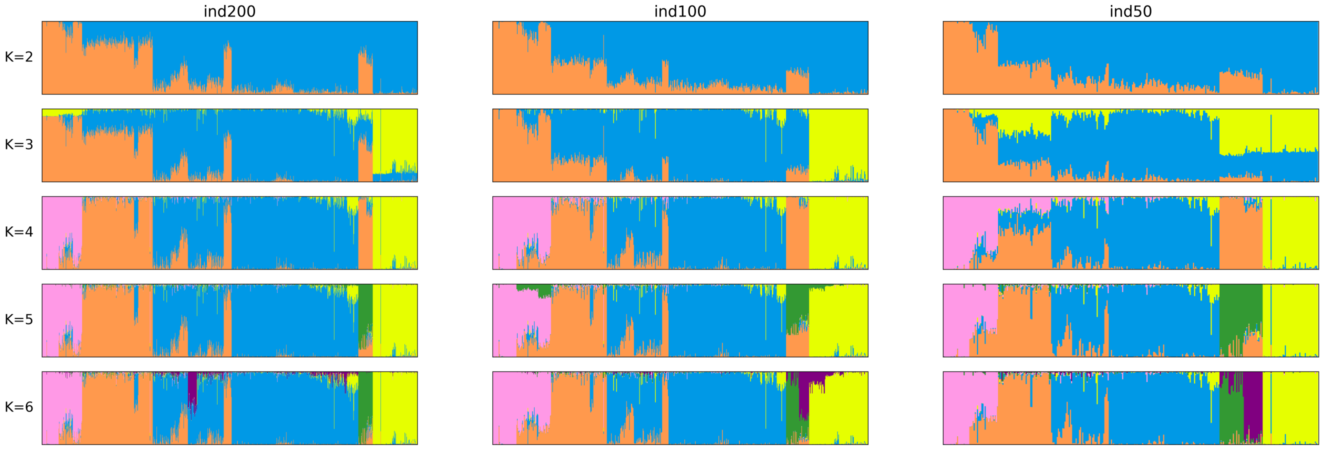

Figure 13: Aligned modes for population structure inferred from subsets of different sample sizes in HGDP data: (left) 200 individuals from each region; (middle) 100 individuals from each region; (right) individuals from each region.

9 Customizable Algorithms

In addition to carefully choosing the parameters, there are several ways to modify *Clumppling*'s framework. For instance, even when the statistical test indicates significant community structure, the detected structure may be too fine-grained if our interest lies in broader-level structural differentiation. To address this, we can either specify a smaller p-value for the community structure test, ensuring that only highly significant community structures are rejected, or introduce additional quality check procedures after community detection to prevent overly similar modes from being separated.

The choice of community detection method also influences alignment performance. Apart from the Louvain algorithm, there are numerous other community detection methods available, and each method may exhibit different behavior in mode detection tasks. *Clumppling* can be easily modified to incorporate alternative community detection methods.

Changing *Clumppling* to include customized algorithms requires the modification of provided Python code files. If you install the package via downloading the package files, you can find the code files under the "Clumppling/-clumppling" folder. If you install the package directly using git, you may locate your package files by

```
pip show clumppling
```

and then navigate to the location shown.

9.1 Custom community detection method

To use a custom community detection method, you need to modify the code file "custom_funcs.py" to implement the customized community detection function `cd_custom` and change the corresponding parameter in the parameter file. Once you locate the file, make changes to the following function:

```
def cd_custom(G):
    """Customized community detection method (need to be modified)

    Parameters
    ----------
    G : networkx.Graph
        the similarity network of replicates, with edges weighted by similarity after optimal alignment

    Returns
    -------
    partition_map
        a dictionary where keys are the indices of replicates and values are the indices of the communities they belong to

    Please comment out the dummy placeholder and customize your community detection method there.
    Two examples are provided below in commented text.
    """

    # Please comment out the following line and customize your community detection method here.
    ## Dummuy placeholder
    partition_map = {i:0 for i in range(G.number_of_nodes())}

    return partition_map
```

Figure 14: Customizable function for community detection in the file "custom_funcs.py".

The `cd_custom` function takes in a NetworkX graph object, where nodes are replicates we want to align, and edges between each pair of replicates are negatively weighted by the optimal alignment cost between them. In general, nodes that are more densely connected (having edges with higher weights among them) should belong to the same community, while nodes that are connected by weaker edges (those with lower weights) should belong to different communities. The function should output a Python dictionary object containing the information about partitioning the nodes into communities.

The current function provided contains a dummy line of assigning all nodes (replicates, indexed starting from 0) into one community (mode, labeled 0). You should comment out this dummy line and implement your own community detection method that gives a valid partition map. You may refer to our default Louvain method implemented in the `cd_default` function in the file "funcs.py" as an example.

Next, reinstall the program by calling "`pip install -e .`" again, then you can run *Clumppling* with your customized community detection method for mode detection by setting the parameter `"cd_default"` to `0` in input arguments.

9.2 Alternative p-value for testing the existence of community structure

The default p-value threshold used for testing the existence of community structure is $p = 0.01$. If you want to use a different p-value, you may change one line in the file "funcs.py":

Locate the following line, which is in the function `detect_modes()` in the function file,

```
has_comm_struc = test_comm_struc(adj_mat, alpha = 0.01)
```

and change the value 0.01 to the specific p-value you want to use.

Next, reinstall the program by calling "`pip install -e .`", then you can run *Clumppling* with your customized community structure test.

If you do not want to download the package and run it locally, we also offer the option to use it online directly via a Google Colaboratory notebook at `https://colab.research.google.com/drive/1PiM5pUKm9cx-dCzOYLWwaJcNcTQHyUm8#offline=true&sandboxMode=true`.

In order to execute the code, you need to sign in to a Google account. Once you open the notebook, it will automatically create a copy and all the modifications and executions you make will be on this copy. You can also explicitly make a copy of the notebook in Drive.

To run the alignment, you need to prepare a zip file containing all the input files, i.e., the clustering results. Once you have the file ready, simply follow the instructions on the notebook to use *Clumppling*. Click the little round button with an arrowhead on it on the left side of each block to run the block. After clicking through the blocks for installing the package and setting up, upload the input per instruction. Then choose your input data

format, set any optional parameters if you want to, and run the program. Once the program finishes running, you can download the output files that are zipped together in one file. You may also choose to view the output figures directly in the notebook by following the instruction.

References

[1] Aaron A Behr, Katherine Z Liu, Gracie Liu-Fang, Priyanka Nakka, and Sohini Ramachandran. pong: Fast analysis and visualization of latent clusters in population genetic data. *Bioinformatics*, 32(18):2817–2823, 2016.

[2] Alyssa Lyn Fortier, Jaehee Kim, and Noah A Rosenberg. Human-genetic ancestry inference and false positives in forensic familial searching. *G3: Genes, Genomes, Genetics*, 10(8):2893–2902, 2020.

[3] Naama M Kopelman, Jonathan Mayzel, Mattias Jakobsson, Noah A Rosenberg, and Itay Mayrose. Clumpak: a program for identifying clustering modes and packaging population structure inferences across k. *Molecular ecology resources*, 15(5):1179–1191, 2015.

[4] Jonathan K Pritchard, Matthew Stephens, and Peter Donnelly. Inference of population structure using multilocus genotype data. *Genetics*, 155(2):945–959, 2000.

[5] Sohini Ramachandran, Noah A Rosenberg, Lev A Zhivotovsky, and Marcus W Feldman. Robustness of the inference of human population structure: a comparison of x-chromosomal and autosomal microsatellites. *Human genomics*, 1(2):1–11, 2004.

[6] Noah A Rosenberg, Terry Burke, Kari Elo, Marcus W Feldman, Paul J Freidlin, Martien AM Groenen, Jossi Hillel, Asko Mäki-Tanila, Michele Tixier-Boichard, Alain Vignal, et al. Empirical evaluation of genetic clustering methods using multilocus genotypes from 20 chicken breeds. *Genetics*, 159(2):699–713, 2001.

[7] Noah A Rosenberg, Jonathan K Pritchard, James L Weber, Howard M Cann, Kenneth K Kidd, Lev A Zhivotovsky, and Marcus W Feldman. Genetic structure of human populations. *science*, 298(5602):2381–2385, 2002.

[8] Paul Verdu, Ethan M Jewett, Trevor J Pemberton, Noah A Rosenberg, and Marlyse Baptista. Parallel trajectories of genetic and linguistic admixture in a genetically admixed creole population. *Current Biology*, 27(16):2529–2535, 2017.

[9] Naama M. Kopelman Xiran Liu and Noah A. Rosenberg. A dirichlet model of alignment cost in mixed-membership unsupervised clustering. *Journal of Computational and Graphical Statistics*, 32(3):1145–1159, 2022.
